# Divergent ancestry of Korean native and Thai chickens with independent gene pool retention by Korean commercial chickens

**DOI:** 10.5713/ab.25.0315

**Published:** 2025-10-22

**Authors:** Ekerette Ekerette, Trifan Budi, Chien Phuoc Tran Nguyen, Nichakorn Kumnan, Worapong Singchat, Wongsathit Wongloet, Piangjai Chalermwong, Anh Huynh Luu, Thitipong Panthum, Aingorn Chaiyes, Kanithaporn Vangnai, Chotika Yokthongwattana, Chomdao Sinthuvanich, Narongrit Muangmai, Prateep Duengkae, Dong-Yep Oh, Kyudong Han, Seyoung Mun, Kornsorn Srikulnath

**Affiliations:** 1 Animal Genomics and Bioresource Research Unit (AGB Research Unit), Faculty of Science, Kasetsart University, Bangkok, Thailand; 2 Animal Genetics and Genomic Unit, Department of Genetics and Biotechnology, University of Calabar, Calabar, Nigeria; 3 Interdisciplinary Graduate Program in Bioscience, Faculty of Science, Kasetsart University, Bangkok, Thailand; 4 Special Research Unit for Wildlife Genomics (SRUWG), Department of Forest Biology, Faculty of Forestry, Kasetsart University, Bangkok, Thailand; 5 The International Undergraduate Program in Bioscience and Technology, Faculty of Science, Kasetsart University, Bangkok, Thailand; 6 Department of Food Science and Technology, Faculty of Agro-Industry, Kasetsart University, Bangkok, Thailand; 7 Department of Biochemistry, Faculty of Science, Kasetsart University, Bangkok, Thailand; 8 Department of Fishery Biology, Faculty of Fisheries, Kasetsart University, Bangkok, Thailand; 9 Livestock Research Institute, Yeongju, Korea; 10 Department of Microbiology, Dankook University, Cheonan, Korea; 11 Center for Bio-Medical Engineering Core-Facility, Dankook University, Cheonan, Korea; 12 Smart Animal Bio Institute, Dankook University, Cheonan, Korea; 13 Laboratory of Animal Cytogenetics and Comparative Genomics (ACCG), Department of Genetics, Faculty of Science, Kasetsart University, Bangkok, Thailand

**Keywords:** Gene Pool, Genetic Diversity, Genetic Exchange, Inbreeding, Korean Native Chicken, Microsatellite

## Abstract

**Objective:**

The objective is to investigate the genetic variation and linkages of Korean native chickens (KNCs), which are regarded as a significant genetic resource due to their distinctive features, however remain inadequately studied in these aspects. In this study, we investigated the genetic variation within five KNC varieties: Korean commercial chicken (KOR-C/M), Silkie (KOR-KS), Leghorn (KOR-LH)—all imported and adapted varieties—and two pureline KNCs: Korean traditional Gray Brown (KOR-KGB) and Korean traditional Yellow Brown (KOR-KYB).

**Methods:**

The relationship of KNCs with chicken breeds from Thailand was evaluated using microsatellite genotyping.

**Results:**

High genetic diversity was observed among the KNC breeds, with KOR-KYB showing the highest heterozygosity, whereas KOR-C/M exhibited the lowest heterozygosity and signs of inbreeding. The loci MCW0016, ADL0268, MCW0081, MCW0067, MCW0248, MCW0165, ADL0278, and MCW0330 were identified as being under directional selection. Genetic clustering analyses indicated four distinct clusters, with KOR-C/M differing from other KNC breeds. Distinct gene pools were observed between KNC breeds and indigenous and local chicken populations, as well as red junglefowl in Thailand, suggesting that they have evolved separately. However, connections may exist due to shared ancestry and crossbreeding.

**Conclusion:**

Clustering analysis revealed that KNC breeds formed a separate group from indigenous and local chickens and red junglefowl in Thailand. The distinct genetic patterns suggest independent evolution of the KNC and local Thai breeds, although shared ancestry and crossbreeding may have introduced some connections. These findings bear significant implications for the conservation and sustainable management of KNC populations.

## INTRODUCTION

Progress toward Sustainable Development Goal 2 (zero hunger) remains alarmingly slow, leaving 2.33 billion people worldwide affected by food insecurity [1]. With the global population projected to reach 9.8 billion by 2050 [2], targeted agricultural strategies, such as identifying genetic resources for sustainable farming, are crucial to meet the rising food demand. Compared with other domestic animals, chickens represent a diverse and abundant genetic resource with significant untapped potential for enhancing food security and sustainability [3]. Domesticated from red junglefowl in Thailand during the mid-early Holocene [4], chickens have since developed a wide array of observable traits, such as variations in plumage and comb size, which are often linked to performance characteristics and cultural preferences [5]. Chickens are now globally widespread, and their genetic diversity reflects their extensive history of dispersal and adaptation to numerous environments [4]. Commercial, indigenous, and local breeds are considered valuable genetic resources for analyzing the relationships between traits and genes, as well as the effects of selection on genomic diversity [6,7]. Indigenous and local chickens are recognized for their adaptability to specific local environments and are considered vital for sustainable poultry production [4]. This aspect is of great interest for studies conducted across different regions with varying local environments.

Korean native chickens (KNC) are a breed that originate from the Korean peninsula. A single comb type is displayed by all KNC breeds, and the shanks typically show brown, dark green, or black coloration. The primary distinguishing characteristic among the breeds is the plumage color, which ranges from white, black, yellowish-brown, greyish-brown, to reddish-brown. These color variations have been used as the basis for their classification [8]. The mature body weight is 2.5 to 3.1 kg for males and 1.6 to 2.0 kg for females. Hens produce light brown eggshells [9]. Domestication of the KNC breed occurred approximately 2,000 years ago, leading to the development of distinct breeds in geographically isolated regions, primarily differentiated by plumage coloration [8,9]. However, the KNC population was substantially reduced to the point of near extinction after the Korean War because of the importation of exotic breeds and the commercialization of these imported breeds. In late 1970s, the National Institute of Animal Science (NIAS) in Korea launched a project on the maintenance and conservation of the KNC breed [8–10]. Consequently, five KNC breeds were established based on feather color, body shape, and external appearance. These breeds, namely, White (Baeksaek Jarae-jong), Black (Heuksaek Jaerae-jong), Grey (Hoegalsaek Jaerae-jong), Yellow (Hwanggalsaek Jaerae-jong), and Red KNC (Jeokgalsaek Jaerae-jong) have been registered with the Domestic Animal Diversity Information Service (DAD-IS) of Food and Agriculture Organization of the United Nations (FAO) [8]. Two types of purebred chicken lines have been preserved by NIAS: the KNC breed and “imported and adapted” breeds, such as Silkie and Leghorn. These imported breeds, recognized for their strong productivity traits, were introduced in the 1960s for industrialization and were also utilized to enhance the productivity of the KNC breed, particularly in the earlier stages of breeding [8–10]. Because of the similar morphological features of these breeds and the reliance of breeding strategies on plumage color, it was assumed that the KNC and imported breeds might share similar genetic characteristics. This genetic similarity could result in the loss of unique traits over time and increase vulnerability to diseases and environmental changes [8].

Assessing the unique genetic identity of the KNC breed requires looking beyond the relatively recently documented crosses within Korea. Understanding its deeper historical origins and potential genetic exchange with other breeds is also crucial. Historically, collaborative exchanges have existed between Thailand and Korea through trade partnerships. Currently, strong bilateral ties are maintained between Korea and Thailand, supported by significant trade facilitated by Asia-Pacific Economic Cooperation (APEC) membership and various Memoranda of Understanding [11]. This active economic exchange likely includes the chicken trade. If the KNC breed originated in Thailand, the genetic characteristics of chicken populations in both regions may have been shaped by shared origins and ongoing trade [12]. Therefore, the genetic footprint of indigenous and local chicken breeds and/or red junglefowl in Thailand might be present in the KNC breed. In this study, the genetic diversity, structure, and relationships between Korean and Thai chickens were assessed to identify valuable poultry genetic resources. Evaluations were made among KNC breeds (Gray-Brown and Yellow-Brown), Korean commercial chickens, and imported breeds (Silkie and Leghorn) using 28 microsatellite markers and compared with gene pool data from Thai chicken breeds and red junglefowl under the Siam Chicken Bioresource Project (SCBP) [13–16]. The results of the genetic diversity and relationships between Korean and Thai chickens are expected to provide potential insights for informed strategies in sustainable poultry breeding.

## MATERIALS AND METHODS

### Specimen collection and DNA extraction

Blood specimens were collected from 315 Korean chickens from five populations: Korean commercial chickens (KOR-C/M; n = 240), Silkie (KOR-KS; n = 17), Korean traditional chickens (Gray Brown) (KOR-KGB; n = 25), Korean traditional chickens (Yellow Brown) (KOR-KYB; n = 17), and Leghorn (KOR-LH; n = 16), as presented in Supplement 1. All Korean chicken samples were raised and preserved in Gyeongsangbuk-do Province, Republic of Korea. Individuals were randomly selected from available populations, ensuring that no two chickens were from the same immediate family to reduce relatedness bias. Sex of the sampled individuals was not considered in the study, as the genetic analyses conducted were independent of sex-linked variation. Specimen collection, DNA extraction, and DNA qualification and quantification were performed as described previously [13]. Briefly, blood specimen collections were collected from the chicken wing vein followed by DNA extraction using a standard salting-out protocol. DNA qualification and quantification were assessed by 1% agarose gel electrophoresis and a NanoDrop 2000 Spectrophotometer (Thermo Fisher Scientific).

### Microsatellite genotyping and data analysis

Twenty-eight microsatellite primer sets based on the FAO recommendations were used for genotyping to assess the biodiversity of chicken populations [17]. The 5′-end of each forward primer was labeled with either 6-fluorescein amidite (6-FAM) or hexachlorofluorescein (HEX) (Macrogen). Microsatellite PCR amplification was performed in triplicate for each sample, following the method described previously under “Siam Chicken Bioresource Project (SCBP)” to ensure accurate results and minimize the chance of false allele amplification [13–16]. Genetic diversity was assessed by calculating the observed and expected heterozygosity (*H*_o_ and *H*_e_), allelic richness (*AR*), polymorphic information content (*PIC*), number of alleles per population (*N*_a_), *F*-statistics (*F*_IS_ and *F*_ST_), relatedness (*r*), and pairwise Nei’s genetic distance in GenAlex [18] as previously described by Budi et al [13]. Welch’s t-test, implemented in R, was used to compare the p-value between *H*_e_ and *H*_o_. Hardy-Weinberg equilibrium (*HWE*) and linkage disequilibrium (LD) were evaluated using Arlequin software ver. 3.5.2.2 [19]. Analysis of molecular variance (AMOVA) was conducted using Arlequin ver. 3.5.2.2 to identify the group structures [19]. The allelic range was obtained for each locus using Arlequin ver. 3.5 [19], which was used to calculate the relative long-term bottleneck events based on the *M* ratio. The Wilcoxon signed-rank test for detecting recent population bottlenecks was performed using a two-phase mutation model (TPM) and stepwise mutation model (SMM) to assess the probability of excess heterozygosity due to small sample sizes. A selective sweep analysis was performed, in which the *H*_e_ and *F*_IS_ values of the chicken populations were plotted for each microsatellite locus (28 loci in total) and five KNC breeds, as previously described [13]. High *F*_IS_ and low *H*_e_ values reflect sweeping or purifying/negative selection, whereas low *F*_IS_ and high *H*_e_ values indicate neutral or balanced selection [20]. Microsatellite locus neutrality was assessed using the Bayesian regression approach in BAYESCAN [21], which calculates the Bayes factor to estimate the probability of locus selection. Based on these data, this factor represents the ratio of the posterior probabilities of the two models, selection and neutral.

To explore the relationships among individual chickens of different varieties and populations, principal coordinate analysis (PCoA) was performed using GenAlEx ver. 6.5 [18]. The ADEGENET package in R ver. 4.3.2 [22] was used to perform the discriminant analysis of principal components (DAPC). Factorial correspondence analysis (FCA) was performed using GENETIX ver. 4.05, based on allelic frequency data [23]. Additionally, multidimensional scaling (MDS) analysis was performed in Python ver. 3.7.1, using the MDS function from the sklearn.manifold submodule to assess individual sample differences. Population structure analysis was conducted using a model-based clustering approach in STRUCTURE ver. 2.3.4 [24]. The analysis involved 100,000 Markov chain Monte Carlo repetitions after a burn-in period of 100,000 generations, using correlated allelic frequencies under a standard admixture model. The number of clusters (*K*) varied from 1 to 25 with 15 repetitions for each value of *K*. The optimal number of clusters was determined by plotting the log-likelihood of the data (ln Pr (X|*K*) across different *K* values and selecting the point at which it stabilized. A structural Selector was used to implement the Δ*K* method [25]. The potential origin of the Korean chicken was assessed using Approximate Bayesian Computation (ABC) analysis implemented in DIYABC ver. 2.1.0 [26], which was used to explain the statistical origin of Korean chickens by estimating the posterior probabilities of past scenarios. Genotypic data generated in this study were stored in the Dryad Digital Repository dataset ( https://doi.org/10.5061/dryad.hhmgqnkm0; accessed on 30 April 2025).

### Investigation of the genetic relationship among Korean chicken varieties, red junglefowl, and other indigenous and local Thai chickens

The genetic relationship between Korean chicken varieties, the red junglefowl, indigenous chickens, and local Thai chicken breeds was examined using microsatellite genotyping data from various chicken populations, which are available through the SCBP [13–16]. All indigenous and local chicken populations were treated as separate populations. Pairwise genetic distances between populations and clustering analyses based on PCoA, DAPC, FCA, MDS, and the model-based clustering approach developed in STRUCTURE ver. 2.3.4, were used following Budi et al [13].

## RESULTS

### Genetic diversity of the Korean chicken varieties based on microsatellite genotyping data

A total of 1,260 alleles were observed in the five Korean chicken varieties, with the mean number of alleles per locus as 3.984±1.487 (Table 1 and Supplement 2). Most allelic frequencies showed significant departure from Hardy–Weinberg equilibrium, with multiple lines of evidence for LD (Supplement 2). Null alleles were found at the MCW0165 locus; nevertheless, all markers were treated similarly. F statistics were negative for KOR-KS, KOR-KGB, KOR-KYB, and KOR-LH. By contrast, KOR-C/M exhibited a positive F statistic. The overall mean F value was −0.098±0.087 (Table 1). The *PIC* ranged from 0.517 to 0.761, with an average of 0.604±0.042, whereas *I* was from 1.092 to 1.934 with an average of 1.355± 0.149 (Table 1). The mean *H*_o_ and *H*_e_ values were 0.695±0.026 and 0.652±0.036, respectively (Table 1). The mean *N*_ea_ of the alleles for the Korean chicken varieties was 3.713±0.522, while the mean *AR* value was 7.002±2.147. The *M* ratios ranged from 0.176 to 0.261 with a mean of 0.230±0.015 which was lower than the 0.68 threshold by Garza and Williamson [27], suggesting a rapid historical population decline (Table 1). The standard genetic diversity parameters per population are summarized in Table 1, and Supplement 2 presents detailed information on the genetic diversity parameters per microsatellite locus. Welch’s t-test revealed significant differences (p*<*0.05) between *H*_o_ and *H*_e_ in KOR-C/M and KOR-KS, whereas KOR-KGB, KOR-KYB, and KOR-LH were not significantly different (Supplement 3). A pairwise comparison of genetic diversity parameters revealed significant differences in *H*_o_ between KOR-C/M and KOR-KS, KOR-C/M and KOR-KGB, and KOR-C/M and KOR-KYB (Supplement 4). Similarly, there were significant differences (p*<*0.05) in *H*_e_ between KOR-C/M and KOR-KS, KOR-C/M and KOR-KGB, KOR-C/M and KOR-KYB, and KOR-C/M and KOR-LH groups. The average *F*_IS_ values for KOR-C/M, KOR-KS, KOR-KGB, KOR-KYB, and KOR-LH were 0.226, −0.262, −0.161, −0.191, and −0.169, respectively, while the mean *r* values were −0.003, −0.022, −0.030, −0.030, and −0.034, respectively (Supplement 5). The pairwise distribution of *r* was significant (p<0.05) for the majority of the populations, except for KOR-C/M and All population, KOR-KS and KOR-KGB, KOR-KS and KOR-LH, KOR-KGB and KOR-KYB, KOR-KGB and KOR-LH, and KOR-KYB and KOR-LH (Supplement 6). All population combinations significantly affected the distribution of *F*_IS_ except for KOR-KGB, KOR-LH, KOR-KYB, and KOR-LH (Supplement 6). The *F*_ST_ value was significant (p*<*0.05) for pairwise comparisons among all varieties after 110 permutations (Supplement 7). AMOVA based on the 28 microsatellite loci revealed that 86.45% of the variation was within varieties, whereas 13.55% was among varieties (Supplement 8). From the Nei genetic distance, the highest pairwise genetic distance of 0.619 was observed between KOR-KS and KOR-LH while the lowest distance of 0.252 was between KOR-KS and KOR-KGB (Supplement 9).

Population clustering analysis using PCoA, DAPC, FCA, and MDS revealed that the five Korean chicken varieties could be divided into four distinct clusters, three of which consisted primarily of KOR-C/M members. The KOR-KS, KOR-KGB, KOR-KYB, and KOR-LH members formed a single cluster, with some KOR-C/M members also included in this group (Supplements 10–13). Different gene pool patterns were observed between populations based on model-based Bayesian algorithms implemented in STRUCTURE with increased *K*-values (*K* = 1 to *K* = 25). The highest posterior probability was obtained at *K* = 3 based on Evanno’s Δ*K*, while the mean ln P(*K*) showed the highest probability at *K* = 16 (Supplement 14). At *K* = 3, KOR-C/M exhibited two unique gene pool patterns. By contrast, KOR-KS, KOR-KGB, KOR-KYB, and KOR-LH showed similar gene pool patterns, although different gene pools were found in KOR-C/M. Traces of KOR-C/M were also found in the KOR-LH gene pool. At the highest ln P(*K*) value (*K* = 16), KOR-C/M exhibited multiple gene pool patterns. Traces of KOR-C/M were also found in the KOR-LH gene pool. By contrast, the KOR-KS and KOR-KYB strains exhibited distinct unique gene pools. KOR-KGB comprised gene pools from both KOR-KS and KOR-KYB. KOR-LH contained two gene pool patterns, one unique to KOR-LH and the other derived from KOR-KYB. A similar trend was observed at *K* = 19. At the highest *K*-value (*K* = 25), KOR-C/M exhibited multiple gene pool patterns. By contrast, KOR-KS had a unique gene pool pattern, whereas KOR-KGB had two gene pool patterns derived from KOR-KS and KOR-KYB. The KOR-LH gene pool was also detected in KOR-KYB at this *K*-value (Supplement 15). ABC analysis identified Scenario 3 as having the highest posterior probability (0.8971), suggesting that the Korean chicken varieties likely originated from a common ancestor. This ancestor split with one branch becoming KOR-LH retained its original characteristics over time (Supplement 16). The other group exhibited changing characteristics, leading to the formation of KOR-KYB and subsequently KOR-KGB. Over time, KOR-C/M, which shares similar traits with KOR-KYB, was isolated from KOR-KS. Meanwhile, Scenarios 1 (0.0009) and 2 (0.1019) exhibited very low posterior probabilities.

The genetic selective sweep plot revealed higher *H*_e_ values than *F*_IS_ in the five Korean chicken varieties, indicating neutral or balanced selection (Supplement 17). Similarly, the 28 microsatellite loci also revealed a higher *H*_e_ than *F*_IS_ indicating neutral or balanced selection, except for locus ADL278, which was under sweeping or purifying selection (Supplement 17). Loci MCW0016, ADL0268, MCW0081, MCW0067, MCW0248, MCW0165, ADL0278, and MCW0330 were identified as likely loci under directional selection (Supplement 18). The Wilcoxon signed-rank test ranged from 0.124 to 1.000 and 0.214 to 1.000 for TPM and SMM, respectively, with a normal L-shaped mode shift, indicating the absence of a recent bottleneck in Korean chicken varieties (Supplement 19). All the calculation results are available from the Dryad Digital Repository Dataset ( https://doi.org/10.5061/dryad.hhmgqnkm0; accessed on 30 April 2025).

### Genetic relationship among Korean chickens, red junglefowl, and other indigenous and local chicken varieties in Thailand

**Figure 1:**
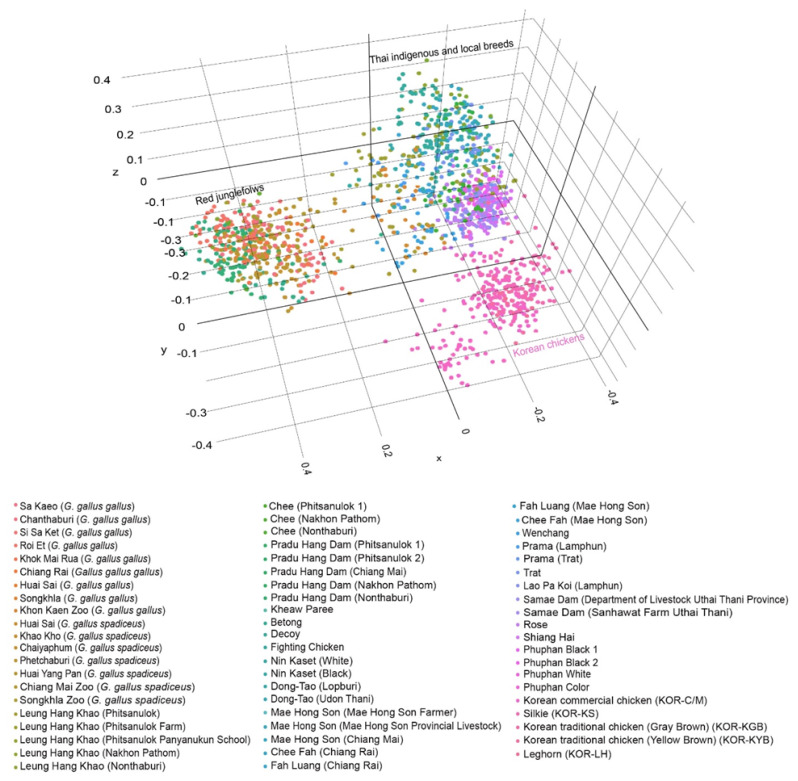
Principal coordinate analysis (PCoA) of Korean chicken varieties, red junglefowl, and Thai domestic chicken breeds based on 28 microsatellite loci. Different populations/breeds are represented by different colors.

**Figure 2:**
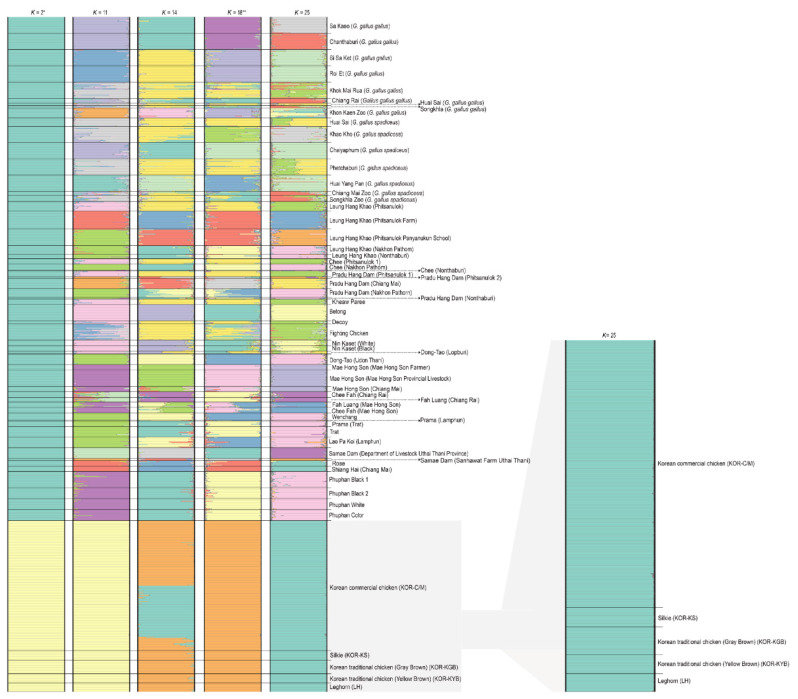
Population structure of five Korean chicken varieties with red junglefowl and domestic breeds in Thailand. The *x*-axis represents the proportion of membership (posterior probability) in each genetic cluster, while each horizontal bar on the *y*-axis represents an individual. All individuals from the five varieties are superimposed on the plot. Black vertical lines indicate the boundaries. The highest posterior probability, denoted by *, was determined based on Evanno’s Δ*K*, and the highest ln P(*K*) is represented by **.

AMOVA analysis of Korean chickens with those from other indigenous and local chicken varieties and red junglefowl in Thailand revealed a higher variation within the population (73.15%) than among populations (26.85%). The PCoA grouped the chicken populations into three clusters (Figure 1). All five Korean chicken varieties belonged to a single cluster. Similarly, all the species of red junglefowl such as Sa Kaeo (*G. gallus gallus*), Chanthaburi (*G. gallus gallus*), Huai Yang Pan (*G. gallus spadiceus*), Khao Kho (*G. gallus spadiceus*), Roi Et (*G. gallus gallus*), Si Sa Ket (*G. gallus gallus*), Chaiyaphum (*G. gallus spadiceus*), Phechaburi (*G. gallus spadiceus*), and Khok Mai Rua (*G. gallus gallus*) were in the same cluster while all the indigenous and local Thai breeds were clustered together. The DAPC results (Supplement 20) also group the chickens into three clusters, conforming with the results of the FCA and MDS. STRUCTURE analysis revealed distinct gene pool patterns with increase in *K*-values ranging from *K* = 1 to *K* = 25 (Figure 2). The highest posterior probability, based on Evanno’s Δ*K* was obtained at *K* = 2, whereas the mean In P(*K*) had the highest peak at *K* = 18. At *K* = 2, the five Korean chicken breeds had a distinct gene pool pattern, separating them from Thai chicken breeds, including red junglefowl. At *K* = 14, some gene pool patterns of KOR-C/M were observed in KOR-KS, KOR-KGB, KOR-KYB, and KOR-LH. Additionally, the gene pool of KOR-C/M was similar to Thai Phuphan black bone chickens (Phuphan black 1, Phuphan black 2, Phuphan white, and Phuphan color), Lueng Hang Khao (Nakhon Pathom), Lueng Hang Khao (Nonthaburi), Chee (Nakhon Pathom), Prama (Trat), Trat, as well as four red junglefowl from Sa Kaeo (*G. gallus gallus*), Chanthaburi (*G. gallus gallus*), Chiang Rai (*G. gallus gallus*), and Chiayaphum (*G. gallus gallus*) in Thailand. At *K* = 18, all the Korean chicken breeds shared similar gene pool patterns. By contrast, Thai chickens showed gene pool sharing across different breeds. The Phuphan black-bone chicken exhibited a gene pool pattern similar to that of breeds such as Trat, Prama (Trat), Mae Hong Son (Chiang Mai), Chee Fah (Chiang Rai), Chee (Nakhon Pathom), Leung Hang Khao (Nonthaburi), and Leung Hang Khao (Nakhon Pathom). Additionally, Sa Kaeo (*G. gallus gallus*) and Chanthaburi (*G. gallus gallus*) displayed similar gene pool patterns. Moreover, Si Sa Ket (*G. gallus gallus*) and Roi Et (*G. gallus gallus*) also exhibited similar gene pool patterns. At the highest *K*-value (*K* = 25; KOR-KGB), all Korean chickens continued to exhibit similar gene pool patterns, which were also comparable to the gene pool patterns of Rose and Shiang Hai chickens from Thailand. The detailed results of all the calculations are available in the Dryad Digital Repository (https://doi.org/10.5061/dryad.hhmgqnkm0; accessed on 30 April 2025).

## DISCUSSION

Preserving high genetic diversity is critical to foster the adaptive capacity and population persistence to ensure the sustainability of poultry production systems [6,7]. The KNC breeds used in this study displayed a considerably high genetic diversity. The highest genetic diversity was found in KOR-KYB, whereas KOR-C/M exhibited the lowest. The magnitude of genetic diversity of these varieties was comparable to that reported in previous studies [9,28–31]. Notably, KOR-C/M exhibited signs of inbreeding, as evidenced by reduced *H*_o_ relative to *H*_e_, coupled with a positive *F*_IS_ value. As a commercial variety, KOR-C/M has likely undergone strict selective breeding to maintain desirable traits, such as egg or meat production [31], a practice that may have progressively restricted genetic diversity. Over time, this may have resulted in a closed breeding population and increased homozygosity [32]. The potential selective breeding effect on inbreeding in KOR-C/M genetic diversity is further supported by significant deviations from the Hardy-Weinberg equilibrium, as shown by the HWE test [33]. By contrast, the KNC breeds (KOR-KS, KOR-KGB, KOR-KYB, and KOR-LH) potentially managed in an effective manner to retain low inbreeding levels and considerably stable genetic diversity over time [9,28–31]. However, a high-density single nucleotide polymorphism (SNP) array study reported unexpected inbreeding signals in KNC breeds rather than in commercial breeds [10], which was potentially influenced by sampling bias due to uneven population representation in the study [34]. This disparity may also have arisen from the use of different molecular markers. SNP-based analyses may sometimes provide biased estimates of low genetic diversity compared with traditional microsatellite markers [35]. Future investigations should prioritize balanced sampling across KNC subpopulations, and high-throughput SNP genotyping, such as whole-genome sequencing, should be employed to clarify the inbreeding dynamics within KNC breeds [36].

The KNC breeds may have experienced historical bottlenecks, as indicated by the Wilcoxon signed-rank test and *M*-ratio test. These results may align with the near-extinction of the KNC during the mid-20th century, which was driven by minimal commercialization and disruptions caused by the Korean War [8]. Postwar conservation efforts have led to renewed interest in breed improvement, resulting in the establishment of structured breeding programs to mitigate genetic loss and preserve KNC lineages [8–10]. These initiatives led to the successful restoration of KNC populations by the NIAS in 2008 [8–10]. Subsequent genetic analyses have confirmed that the diversity within these breeds has remained relatively stable over time [9,28–31].

### Patterns of genetic variation in Korean chicken varieties

Clustering analyses based on PCoA, DAPC, FCA, and MDS revealed four distinct genetic clusters among Korean chicken varieties. The KOR-C/M group displayed pronounced divergence and separated into three distinct clusters, whereas KOR-KS, KOR-KGB, KOR-KYB, and KOR-LH were grouped into a single cluster. This suggests that closer genetic relationships exist among the KOR-KS, KOR-KGB, KOR-KYB, and KOR-LH varieties than among the commercial varieties. This suggests that the observed clustering, which reflects subpopulation structure, is likely caused by the use of multiple genetically distinct yet individually low-diversity lines in commercial breeding rather than by high overall genetic variation. Alternatively, KOR-C/M comprises several genetically differentiated subgroups, each of which has limited genetic diversity, resulting in low overall diversity estimates. This was further supported by the STRUCTURE results, which demonstrated that KOR-C/M maintained a distinctive gene pool pattern compared to KOR-KS, KOR-KGB, KOR-KYB, and KOR-LH across different *K*-values. Similarly, a single cluster consisting of various KNC breeds and multiple clusters from commercial varieties have also been observed based on high-density SNP genotyping [10]. For the KNC breeds (KOR-KGB and KOR-KYB) developed for consumption (meat and egg production) [8], genetic clustering patterns are often influenced by breeding strategies, resulting in the placement of these varieties into a single cluster [10]. Despite the presence of imported and adapted breeds such as Silkie (KOR-KS) and Leghorn (KOR-LH), KOR-KGB and KOR-KYB were also clustered with them. KOR-KS, which grouped closely with KOR-KGB, and KOR-KYB, which clustered with KOR-LH, may reflect historical crossbreeding between indigenous KNCs and imported breeds during breeding programs aimed at improving productivity [10]. Alternatively, this pattern may indicate shared ancestral origins before breed formalization, as supported by mtDNA D-loop data [37]. Although KOR-KGB and KOR-KYB are recognized as pure line KNCs, these findings suggest that their genetic makeup, which may include contributions from imported breeds, warrants further investigation into their breeding history. By contrast, internal gene pool mixing was observed in KOR-C/M, but the same gene pool was not shared with the other KNC breeds. This pattern is likely attributable to multibreed crossbreeding, suggesting that KOR-C/M is maintained independently from other KNC breeds [10]. However, some individuals from KOR-C/M were also grouped with other chicken breeds, suggesting that crossbreeding with KNC breeds may have occurred during the development of KNC breeds to improve productivity. This may also reflect the existence of genetically differentiated subgroups within the KOR-C/M variety.

KNCs are primarily distinguished by their feather color, especially during development [8–10]. This practice has resulted in desirable culinary traits, such as a flavorful taste, lean muscle composition, and firm texture, which are preferred by consumers over broilers [31]. Specific genes or genomic regions associated with these traits can be identified using bioinformatic approaches [6,7]. Selective pressure on these genes may lead to a hitchhiking effect, influencing the allele frequencies of closely linked, selectively neutral loci such as microsatellites [38]. Plumage color is influenced by *RAB17*, *MLPH*, *GRM5*, *SOX5*, and *EGR1*, which are located on chromosomes 1, 3, 4, 8, and 12 respectively [39], whereas our microsatellite markers were also located on 14 chromosomes (chromosomes 1, 2, 3, 4, 5, 6, 7, 8, 10, 13, 14, 17, 23, and 26) [17]. Additionally, genes related to growth and meat quality traits, such as *PIT1*, *ODC*, *GALNTL6*, *SCP2*, and *PLCH2*, are located on chromosomes 1, 3, 4, 8, and 21, respectively [7]. Specifically, the loci MCW0016, ADL0268, MCW0081, MCW0067, MCW0248, MCW0165, ADL0278, and MCW0330 provided evidence of the hitchhiking effect. Notably, the locus ADL0278 also showed evidence of a selective sweep. This locus is located on chromosome 8 and is potentially linked to genes that encode proteins related to carcass weight and egg aftertaste [7], as well as *LEPR* gene, which plays a regulatory role in ovarian follicle development and egg laying [40]. This observation suggests a potential link between these microsatellite loci and the genomic regions selected for plumage color and growth traits in KNC breeds. However, these findings may be coincidental; therefore, further investigations using whole-genome sequencing are needed to clarify the genetic basis of phenotypic traits.

### Genetic relationship among Korean chicken varieties, indigenous and local chicken breeds, and red junglefowl in Thailand

Comparing KNC breeds with indigenous and local chicken breeds and red junglefowl in Thailand can inform strategies for sustainable management and conservation of chicken resources. Clustering analysis indicated that KNC breeds, indigenous and local chickens, and red junglefowl in Thailand were independently clustered. Similarly, KNC breeds were previously reported to be distinct from other Asian chicken breeds [28–31]. The distinct genetic differentiation among KNCs, indigenous and local chickens, and red junglefowl in Thailand underscores their unique evolutionary pathways resulting from prolonged local adaptation [3,4]. This process of local adaptation is pivotal in facilitating the adaptation and maintenance of beneficial phenotypic traits, allowing these chicken populations to respond effectively to local climatic conditions and disease challenges [3,4]. Remarkably, similar gene pools were observed in Korean chickens and in a small proportion of local chickens. This suggests that similar breeding strategies for consumption or shared gene pool patterns may indicate a common ancestry or past genetic exchange, as both Thai breeds were imported and adapted to Thailand, as supported by shared D-loop haplotypes [14]. While specific evidence of chicken movements between Korea and Thailand is lacking, potential pathways for genetic exchange could exist because of Southeast Asia’s role as a primary center for chicken domestication and historical trade and cultural exchanges [11,14]. These patterns may coincide. Examining Thai populations, which are genetically and geographically distinct, alongside KNCs provides a broader evolutionary context that is valuable for interpreting the distinctiveness of KNCs and for informing strategies for their sustainable management and conservation. Potential inconsistencies in genotyping methods, which may have arisen from variations in experimental conditions and allele calling criteria despite originating from different studies within the same research laboratory, were addressed by applying standardized allele calling and quality control procedures to harmonize the datasets prior to analysis, following the approaches described in previous studies [5,13,14,38].

## CONCLUSION

This study emphasizes the significant genetic diversity and relationships among KNC breeds and their connections to indigenous and local chickens in Thailand. These findings highlight the importance of understanding historical breeding practices and potential genetic exchange to make informed conservation and sustainable poultry management strategies. Distinct genetic patterns suggest that the KNC and local Thai breeds have evolved separately; however, connections may exist because of shared ancestry and crossbreeding. These insights indicate the need for continued research using advanced genomic techniques such as whole-genome sequencing to further clarify the genetic basis of valuable traits and enhance the conservation of diverse chicken genetic resources. Ensuring the sustainability of poultry production systems is crucial for addressing global food security challenges, and targeted strategies can be developed by leveraging the genetic potential identified in this study.

## Figures and Tables

**Table 1:** Genetic diversity among five varieties of Korean chickens based on 28 microsatellite loci

Varieties		*N*	*N*_a_	*AR*	*N*_ea_	*I*	*H*_o_	*H*_e_	*M*-ratio	*PIC*	*F*	*HWE*
KOR-C/M	Mean	240	15.786	15.531	5.787	1.934	0.598	0.788	0.250	0.761	0.233	***
	SE		1.575	1.546	0.560	0.096	0.030	0.018	0.132	0.021	0.040	
KOR-KS	Mean	17	4.464	3.950	3.032	1.092	0.733	0.575	0.224	0.517	−0.282	ns
	SE		0.572	0.416	0.399	0.101	0.053	0.038	0.107	0.038	0.062	
KOR-KGB	Mean	25	5.714	5.429	3.316	1.292	0.719	0.645	0.238	0.597	−0.125	ns
	SE		0.509	0.459	0.243	0.085	0.049	0.032	0.080	0.033	0.068	
KOR-KYB	Mean	17	5.071	5.003	3.320	1.260	0.743	0.644	0.176	0.594	−0.176	ns
	SE		0.442	0.379	0.276	0.082	0.045	0.031	0.053	0.032	0.062	
KOR-LH	Mean	16	5.179	5.096	3.108	1.195	0.684	0.609	0.261	0.553	−0.140	ns
	SE		0.532	0.515	0.326	0.088	0.054	0.028	0.125	0.031	0.092	
Total	Mean	315	7.243	7.002	3.713	1.355	0.695	0.652	0.230	0.604	−0.098	ns
	SE		2.145	2.147	0.522	0.149	0.026	0.036	0.015	0.042	0.087	

*** p<0.001; ns, not significant.

*N*, Number of samples; *N*_a_, number of alleles; *AR*, allelic richness; *N*_ea_, number of effective alleles; *I*, Shannon’s information index; *H*_o_, observed heterozygosity; *H*_e_, expected heterozygosity; *PIC*, polymorphic information content; *F*, fixation index; *HWE*, Hardy-Weinberg equilibrium; KOR-C/M, Korean commercial chicken; KOR-KS, Silkie; KOR-KGB, Korean traditional chicken (Gray Brown); KOR-KYB, Korean traditional chicken (Yellow Brown); KOR-LH, Leghorn (LH).

## Data Availability

The datasets generated from this study are available in the Dryad Digital Repository (https://doi.org/10.5061/dryad.hhmgqnkm0; accessed on 30 April 2025).

## SUPPLEMENTARY MATERIAL

**Supplement 1**. Representative of Korean chicken specimens used in this study.

**Supplement 2**. Genetic diversity of 315 individuals of Korean chicken varieties based on 28 microsatellite loci.

**Supplement 3**. Observed and expected heterozygosity of Korean chicken varieties based on 28 microsatellite loci.

**Supplement 4**. Comparison of genetic diversity parameters between Korean chicken varieties based on 28 microsatellite loci.

**Supplement 5**. Average *F*_IS_ and relatedness (*r*) among five varieties of Korean chicken.

**Supplement 6**. Distributions of genetic relatedness values (*r*) and inbreeding coefficients (*F*_IS_) for the Korean chicken varieties.

**Supplement 7**. Pairwise comparison of genetic differentiation (*F*_ST_), *F*_ST_^ENA^ values with ENA correction for null alleles, and *R*_ST_ values between five Korean chicken varieties using FSTAT version 2.9.3.

**Supplement 8**. Analysis of molecular variance (AMOVA) for Korean chicken varieties based on 28 microsatellite loci.

**Supplement 9**. Results of pairwise Nei’s genetic distance (*D*) among Korean chicken varieties, based on 28 microsatellite loci.

**Supplement 10**. Principal coordinate analysis (PCoA) of five Korean chickens.

**Supplement 11**. Discriminant Analysis of Principal Components (DAPC) of five Korean chicken varieties.

**Supplement 12**. Factorial Correspondence Analysis (FCA) of five Korean chicken varieties.

**Supplement 13**. Multidimensional Scaling (MDS) of five Korean chicken varieties.

**Supplement 14**. Different population structure patterns of Korean chicken varieties, generated by model-based Bayesian clustering algorithms implemented in STRUCTURE.

**Supplement 15**. Population structure of five Korean chicken varieties.

**Supplement 16**. Assessment of population history models for the Korean chicken varieties using approximate Bayesian computation (ABC) inference.

**Supplement 17**. Mapping of expected heterozygosity (*H*_e_) against inbreeding coefficients (*F*_IS_) along the length of the physical map.

**Supplement 18**. BAYESCAN estimate for the probability of a locus under selection.

**Supplement 19**. Wilcoxon sign rank test to evaluate mutation–drift equilibrium in 315 Korean chickens under different models.

**Supplement 20**. Discriminant Analysis of Principal Components (DAPC) of Korean, Thailand local and indigenous chicken varieties, and red junglefowl.

## References

[1] Food and Agriculture Organization (FAO), International Fund for Agricultural Development (IFAD), United Nations Children’s Fund (UNICEF), World Food Programme (WFP), World Health Organization (WHO) (2024). The state of food security and nutrition in the world 2024: financing to end hunger, food insecurity and malnutrition in all its forms.

[2] United Nations (2023). World population projected to reach 9.8 billion in 2050, and 112 billion in 2100 [Internet].

[3] Tenza T, Mhlongo LC, Ncobela CN, Rani Z (2024). Village chickens for achieving sustainable development goals 1 and 2 in resource-poor communities: a literature review. Agriculture.

[4] Xiang H, Gao J, Yu B (2014). Early holocene chicken domestication in northern China. Proc Natl Acad Sci USA.

[5] Lengkidworraphiphat P, Wongpoomchai R, Taya S, Jaturasitha S (2020). Effect of genotypes on macronutrients and antioxidant capacity of chicken breast meat. Asian-Australas J Anim Sci.

[6] Rubin CJ, Zody MC, Eriksson J (2010). Whole-genome resequencing reveals loci under selection during chicken domestication. Nature.

[7] Huang R, Zhu C, Zhen Y (2024). Genetic diversity, demographic history, and selective signatures of Silkie chicken. BMC Genom.

[8] Jin S, Jayasena DD, Jo C, Lee JH (2017). The breeding history and commercial development of the Korean native chicken. World’s Poult Sci J.

[9] Suh S, Sharma A, Lee S (2014). Genetic diversity and relationships of Korean chicken breeds based on 30 microsatellite markers. Asian-Australas J Anim Sci.

[10] Seo D, Lee DH, Choi N, Sudrajad P, Lee SH, Lee JH (2018). Estimation of linkage disequilibrium and analysis of genetic diversity in Korean chicken lines. PLOS ONE.

[11] The Observatory Economic Complex (OEC) (2022). South Korea-Thailand trade [Internet].

[12] Londe DW, Elmore RD, Davis CA, Hovick TJ, Fuhlendorf SD, Rutledge J (2022). Why did the chicken not cross the road? Anthropogenic development influences the movement of a grassland birds. Ecol Appl.

[13] Budi T, Singchat W, Tanglertpaibul N (2023). Thai local chicken breeds, Chee Fah and Fah Luang, originated from Chinese black-boned chicken with introgression of red junglefowl and domestic chicken breeds. Sustainability.

[14] Hata A, Nunome M, Suwanasopee T (2021). Origin and evolutionary history of domestic chickens inferred from a large population study of Thai red junglefowl and indigenous chickens. Sci Rep.

[15] Singchat W, Chaiyes A, Wongloet W (2022). Red junglefowl resource management guide: bioresource reintroduction for sustainable food security in Thailand. Sustainability.

[16] Tanglertpaibul N, Budi T, Nguyen CPT (2024). Samae Dam chicken: a variety of the Pradu Hang Dam breed revealed from microsatellite genotyping data. Anim Biosci.

[17] Food and Agriculture Organization (FAO) (2001). Report on the state of the world’s animal genetic resources [Internet].

[18] Peakall R, Smouse PE (2012). GenAlEx 6.5: genetic analysis in Excel. Population genetic software for teaching and research: an update. Bioinformatics.

[19] Excoffier L, Lischer HEL (2010). Arlequin suite ver 3.5: a new series of programs to perform population genetics analyses under Linux and Windows. Mol Ecol Resour.

[20] Reddy UK, Abburi L, Abburi VL (2015). A genome-wide scan of selective sweeps and association mapping of fruit traits using microsatellite markers in watermelon. J Hered.

[21] Foll M, Gaggiotti O (2008). A genome-scan method to identify selected loci appropriate for both dominant and codominant markers: a Bayesian perspective. Genetics.

[22] Jombart T (2008). adegenet: a R package for the multivariate analysis of genetic markers. Bioinformatics.

[23] Belkhir K, Borsa P, Chikhi L, Raufaste N, Bonhomme F (2003). GENETIX, genome population interactions, 404. Laboratoire Géme, Population, Interactions.

[24] Pritchard JK, Stephens M, Donnelly P (2000). Inference of population structure using multilocus genotype data. Genetics.

[25] Li YL, Liu JX (2017). StructureSelector: a web-based software to select and visualize the optimal number of clusters using multiple methods. Mol Ecol Resour.

[26] Cornuet JM, Pudlo P, Veyssier J (2014). DIYABC v2.0: a software to make approximate Bayesian computation inferences about population history using single nucleotide polymorphism, DNA sequence and microsatellite data. Bioinformatics.

[27] Garza JC, Williamson EG (2001). Detection of reduction in population size using data from microsatellite loci. Mol Ecol.

[28] Seo DW, Hoque MR, Choi NR (2013). Discrimination of Korean native chicken lines using fifteen selected microsatellite markers. Asian-Australas J Anim Sci.

[29] Seo JH, Oh JD, Lee JH, Seo D, Kong HS (2015). Studies on genetic diversity and phylogenetic relationships of Korean native chicken using the microsatellite marker. Korean J Poult Sci.

[30] Roh HJ, Kim KW, Lee J (2019). Genetic diversity of Korean native chicken populations in DAD-IS database using 25 microsatellite markers. Korean J Poult Sci.

[31] Seo JH, Lee JH, Kong HS (2017). Assessment of genetic diversity and phylogenetic relationships of Korean native chicken breeds using microsatellite markers. Asian-Australas J Anim Sci.

[32] Fu M, Wu Y, Shen J (2023). Genome-wide association study of egg production traits in Shuanglian chickens using whole genome sequencing. Genes.

[33] Yacoub HA, Fathi MM, Al-Homidan IH (2024). Association of ovocalyxin-32 gene variants with egg quality traits in indigenous chicken breeds. Animals.

[34] Meirmans PG (2019). Subsampling reveals that unbalanced sampling affects STRUCTURE results in a multi-species dataset. Heredity.

[35] Panthum T, Ariyaraphong N, Wongloet W (2023). Preserving pure siamese crocodile populations: a comprehensive approach using multi-genetic tools. Biology.

[36] Seeb JE, Carvalho G, Hauser L, Naish K, Roberts S, Seeb LW (2011). Single-nucleotide polymorphism (SNP) discovery and applications of SNP genotyping in nonmodel organisms. Mol Ecol Resour.

[37] Cho CY, Lee PY, Ko YG, Kim HK, Park MN, Yeon SH (2011). Multiple maternal origins of Korean native chicken based on the mtDNA D-loop variation. Korean J Poult Sci.

[38] Budi T, Luu AH, Singchat W (2024). Purposive breeding strategies drive genetic differentiation in Thai fighting cock breeds. Genes Genomics.

[39] Mastrangelo S, Cendron F, Sottile G (2020). Genome-wide analyses identifies known and new markers responsible of chicken plumage color. Animals.

[40] Lei MM, Wu SQ, Li XW, Wang CL, Chen Z, Shi ZD (2014). Leptin receptor signaling inhibits ovarian follicle development and egg laying in chicken hens. Reprod Biol Endocrinol.

