# Supplementary material for: Divergent ancestry of Korean native and Thai chickens with independent gene pool retention by Korean commercial chickens
Source: Anim Biosci. 2025 Oct 22;39(3):250315. doi: 10.5713/ab.25.0315 (PMC12963744; doi:10.5713/ab.25.0315)
Supplement: Supplementary file 1 [file ab-25-0315-Supplementary-1.pdf]

Supplement 1. Representative of Korean chicken specimens used in this study.

No.	Varieties	Sample code	Population	Population Code
1	Commercial	F_C01	Commercial	KOR-C/M
2	Commercial	F_C02	Commercial	KOR-C/M
3	Commercial	F_C03	Commercial	KOR-C/M
4	Commercial	F_C04	Commercial	KOR-C/M
5	Commercial	F_C05	Commercial	KOR-C/M
6	Commercial	F_C06	Commercial	KOR-C/M
7	Commercial	F_C07	Commercial	KOR-C/M
8	Commercial	F_C08	Commercial	KOR-C/M
9	Commercial	F_C09	Commercial	KOR-C/M
10	Commercial	F_C10	Commercial	KOR-C/M
11	Commercial	F_R01	Commercial	KOR-C/M
12	Commercial	F_R02	Commercial	KOR-C/M
13	Commercial	F_R03	Commercial	KOR-C/M
14	Commercial	F_R04	Commercial	KOR-C/M
15	Commercial	F_R05	Commercial	KOR-C/M
16	Commercial	F_R06	Commercial	KOR-C/M
17	Commercial	F_R07	Commercial	KOR-C/M
18	Commercial	F_R08	Commercial	KOR-C/M
19	Commercial	F_R09	Commercial	KOR-C/M
20	Commercial	F_R10	Commercial	KOR-C/M
21	Commercial	F_Y01	Commercial	KOR-C/M
22	Commercial	F_Y02	Commercial	KOR-C/M
23	Commercial	F_Y03	Commercial	KOR-C/M
24	Commercial	F_Y04	Commercial	KOR-C/M
25	Commercial	F_Y05	Commercial	KOR-C/M
26	Commercial	F_Y06	Commercial	KOR-C/M
27	Commercial	F_Y07	Commercial	KOR-C/M
28	Commercial	F_Y08	Commercial	KOR-C/M
29	Commercial	F_Y09	Commercial	KOR-C/M
30	Commercial	F_Y10	Commercial	KOR-C/M
31	Commercial	F_S01	Commercial	KOR-C/M
32	Commercial	F_S02	Commercial	KOR-C/M
33	Commercial	F_S03	Commercial	KOR-C/M
34	Commercial	F_S04	Commercial	KOR-C/M
35	Commercial	F_S05	Commercial	KOR-C/M
36	Commercial	F_S06	Commercial	KOR-C/M
37	Commercial	F_S07	Commercial	KOR-C/M
38	Commercial	F_S08	Commercial	KOR-C/M

No.	Varieties	Sample code	Population	Population Code
39	Commercial	F_S09	Commercial	KOR-C/M
40	Commercial	F_S10	Commercial	KOR-C/M
41	Commercial	F_D01	Commercial	KOR-C/M
42	Commercial	F_D02	Commercial	KOR-C/M
43	Commercial	F_D03	Commercial	KOR-C/M
44	Commercial	F_D04	Commercial	KOR-C/M
45	Commercial	F_D05	Commercial	KOR-C/M
46	Commercial	F_D06	Commercial	KOR-C/M
47	Commercial	F_D07	Commercial	KOR-C/M
48	Commercial	F_D08	Commercial	KOR-C/M
49	Commercial	F_D09	Commercial	KOR-C/M
50	Commercial	F_D10	Commercial	KOR-C/M
51	Commercial	F_H01	Commercial	KOR-C/M
52	Commercial	F_H02	Commercial	KOR-C/M
53	Commercial	F_H03	Commercial	KOR-C/M
54	Commercial	F_H04	Commercial	KOR-C/M
55	Commercial	F_H05	Commercial	KOR-C/M
56	Commercial	F_H06	Commercial	KOR-C/M
57	Commercial	F_H07	Commercial	KOR-C/M
58	Commercial	F_H08	Commercial	KOR-C/M
59	Commercial	F_H09	Commercial	KOR-C/M
60	Commercial	F_H10	Commercial	KOR-C/M
61	Commercial	F_F01	Commercial	KOR-C/M
62	Commercial	F_F02	Commercial	KOR-C/M
63	Commercial	F_F03	Commercial	KOR-C/M
64	Commercial	F_F04	Commercial	KOR-C/M
65	Commercial	F_F05	Commercial	KOR-C/M
66	Commercial	F_F06	Commercial	KOR-C/M
67	Commercial	F_F07	Commercial	KOR-C/M
68	Commercial	F_F08	Commercial	KOR-C/M
69	Commercial	F_F09	Commercial	KOR-C/M
70	Commercial	F_F10	Commercial	KOR-C/M
71	Commercial	F_K01	Commercial	KOR-C/M
72	Commercial	F_K02	Commercial	KOR-C/M
73	Commercial	F_K03	Commercial	KOR-C/M
74	Commercial	F_K04	Commercial	KOR-C/M
75	Commercial	F_K05	Commercial	KOR-C/M
76	Commercial	F_K06	Commercial	KOR-C/M
77	Commercial	F_K07	Commercial	KOR-C/M
78	Commercial	F_K08	Commercial	KOR-C/M
79	Commercial	F_K09	Commercial	KOR-C/M

No.	Varieties	Sample code	Population	Population Code
80	Commercial	F_K10	Commercial	KOR-C/M
81	Commercial	M_Y01	Commercial	KOR-C/M
82	Commercial	M_Y02	Commercial	KOR-C/M
83	Commercial	M_Y03	Commercial	KOR-C/M
84	Commercial	M_Y04	Commercial	KOR-C/M
85	Commercial	M_Y05	Commercial	KOR-C/M
86	Commercial	M_Y06	Commercial	KOR-C/M
87	Commercial	M_Y07	Commercial	KOR-C/M
88	Commercial	M_Y08	Commercial	KOR-C/M
89	Commercial	M_Y09	Commercial	KOR-C/M
90	Commercial	M_Y10	Commercial	KOR-C/M
91	Commercial	M_D01	Commercial	KOR-C/M
92	Commercial	M_D02	Commercial	KOR-C/M
93	Commercial	M_D03	Commercial	KOR-C/M
94	Commercial	M_D04	Commercial	KOR-C/M
95	Commercial	M_D05	Commercial	KOR-C/M
96	Commercial	M_D06	Commercial	KOR-C/M
97	Commercial	M_D07	Commercial	KOR-C/M
98	Commercial	M_D08	Commercial	KOR-C/M
99	Commercial	M_D09	Commercial	KOR-C/M
100	Commercial	M_D10	Commercial	KOR-C/M
101	Commercial	M_R01	Commercial	KOR-C/M
102	Commercial	M_R02	Commercial	KOR-C/M
103	Commercial	M_R03	Commercial	KOR-C/M
104	Commercial	M_R04	Commercial	KOR-C/M
105	Commercial	M_R05	Commercial	KOR-C/M
106	Commercial	M_R06	Commercial	KOR-C/M
107	Commercial	M_R07	Commercial	KOR-C/M
108	Commercial	M_R08	Commercial	KOR-C/M
109	Commercial	M_R09	Commercial	KOR-C/M
110	Commercial	M_R10	Commercial	KOR-C/M
111	Commercial	M_S01	Commercial	KOR-C/M
112	Commercial	M_S02	Commercial	KOR-C/M
113	Commercial	M_S03	Commercial	KOR-C/M
114	Commercial	M_S04	Commercial	KOR-C/M
115	Commercial	M_S05	Commercial	KOR-C/M
116	Commercial	M_S06	Commercial	KOR-C/M
117	Commercial	M_S07	Commercial	KOR-C/M
118	Commercial	M_S08	Commercial	KOR-C/M
119	Commercial	M_S09	Commercial	KOR-C/M
120	Commercial	M_S10	Commercial	KOR-C/M

No.	Varieties	Sample code	Population	Population Code
121	Commercial	M_C01	Commercial	KOR-C/M
122	Commercial	M_C02	Commercial	KOR-C/M
123	Commercial	M_C03	Commercial	KOR-C/M
124	Commercial	M_C04	Commercial	KOR-C/M
125	Commercial	M_C05	Commercial	KOR-C/M
126	Commercial	M_C06	Commercial	KOR-C/M
127	Commercial	M_C07	Commercial	KOR-C/M
128	Commercial	M_C08	Commercial	KOR-C/M
129	Commercial	M_C09	Commercial	KOR-C/M
130	Commercial	M_C10	Commercial	KOR-C/M
131	Commercial	M_H01	Commercial	KOR-C/M
132	Commercial	M_H02	Commercial	KOR-C/M
133	Commercial	M_H03	Commercial	KOR-C/M
134	Commercial	M_H04	Commercial	KOR-C/M
135	Commercial	M_H05	Commercial	KOR-C/M
136	Commercial	M_H06	Commercial	KOR-C/M
137	Commercial	M_H07	Commercial	KOR-C/M
138	Commercial	M_H08	Commercial	KOR-C/M
139	Commercial	M_H09	Commercial	KOR-C/M
140	Commercial	M_H10	Commercial	KOR-C/M
141	Commercial	M_K01	Commercial	KOR-C/M
142	Commercial	M_K02	Commercial	KOR-C/M
143	Commercial	M_K03	Commercial	KOR-C/M
144	Commercial	M_K04	Commercial	KOR-C/M
145	Commercial	M_K05	Commercial	KOR-C/M
146	Commercial	M_K06	Commercial	KOR-C/M
147	Commercial	M_K07	Commercial	KOR-C/M
148	Commercial	M_K08	Commercial	KOR-C/M
149	Commercial	M_K09	Commercial	KOR-C/M
150	Commercial	M_K10	Commercial	KOR-C/M
151	Commercial	M_F01	Commercial	KOR-C/M
152	Commercial	M_F02	Commercial	KOR-C/M
153	Commercial	M_F03	Commercial	KOR-C/M
154	Commercial	M_F04	Commercial	KOR-C/M
155	Commercial	M_F05	Commercial	KOR-C/M
156	Commercial	M_F06	Commercial	KOR-C/M
157	Commercial	M_F07	Commercial	KOR-C/M
158	Commercial	M_F08	Commercial	KOR-C/M
159	Commercial	M_F09	Commercial	KOR-C/M
160	Commercial	M_F10	Commercial	KOR-C/M
161	Commercial	N_G01	Commercial	KOR-C/M

No.	Varieties	Sample code	Population	Population Code
162	Commercial	N_G02	Commercial	KOR-C/M
163	Commercial	N_G03	Commercial	KOR-C/M
164	Commercial	N_G04	Commercial	KOR-C/M
165	Commercial	N_G05	Commercial	KOR-C/M
166	Commercial	N_G06	Commercial	KOR-C/M
167	Commercial	N_G07	Commercial	KOR-C/M
168	Commercial	N_G08	Commercial	KOR-C/M
169	Commercial	N_G09	Commercial	KOR-C/M
170	Commercial	N_G10	Commercial	KOR-C/M
171	Commercial	N_L01	Commercial	KOR-C/M
172	Commercial	N_L02	Commercial	KOR-C/M
173	Commercial	N_L03	Commercial	KOR-C/M
174	Commercial	N_L04	Commercial	KOR-C/M
175	Commercial	N_L05	Commercial	KOR-C/M
176	Commercial	N_L06	Commercial	KOR-C/M
177	Commercial	N_L07	Commercial	KOR-C/M
178	Commercial	N_L08	Commercial	KOR-C/M
179	Commercial	N_L09	Commercial	KOR-C/M
180	Commercial	N_L10	Commercial	KOR-C/M
181	Commercial	N_O01	Commercial	KOR-C/M
182	Commercial	N_O02	Commercial	KOR-C/M
183	Commercial	N_O03	Commercial	KOR-C/M
184	Commercial	N_O04	Commercial	KOR-C/M
185	Commercial	N_O05	Commercial	KOR-C/M
186	Commercial	N_O06	Commercial	KOR-C/M
187	Commercial	N_O07	Commercial	KOR-C/M
188	Commercial	N_O08	Commercial	KOR-C/M
189	Commercial	N_O09	Commercial	KOR-C/M
190	Commercial	N_O10	Commercial	KOR-C/M
191	Commercial	N_W01	Commercial	KOR-C/M
192	Commercial	N_W02	Commercial	KOR-C/M
193	Commercial	N_W03	Commercial	KOR-C/M
194	Commercial	N_W04	Commercial	KOR-C/M
195	Commercial	N_W05	Commercial	KOR-C/M
196	Commercial	N_W06	Commercial	KOR-C/M
197	Commercial	N_W07	Commercial	KOR-C/M
198	Commercial	N_W08	Commercial	KOR-C/M
199	Commercial	N_W09	Commercial	KOR-C/M
200	Commercial	N_W10	Commercial	KOR-C/M
201	Commercial	N_G01	Commercial	KOR-C/M
202	Commercial	N_G02	Commercial	KOR-C/M

No.	Varieties	Sample code	Population	Population Code
203	Commercial	N_G03	Commercial	KOR-C/M
204	Commercial	N_G04	Commercial	KOR-C/M
205	Commercial	N_G05	Commercial	KOR-C/M
206	Commercial	N_G06	Commercial	KOR-C/M
207	Commercial	N_G07	Commercial	KOR-C/M
208	Commercial	N_G08	Commercial	KOR-C/M
209	Commercial	N_G09	Commercial	KOR-C/M
210	Commercial	N_G10	Commercial	KOR-C/M
211	Commercial	N_L01	Commercial	KOR-C/M
212	Commercial	N_L02	Commercial	KOR-C/M
213	Commercial	N_L03	Commercial	KOR-C/M
214	Commercial	N_L04	Commercial	KOR-C/M
215	Commercial	N_L05	Commercial	KOR-C/M
216	Commercial	N_L06	Commercial	KOR-C/M
217	Commercial	N_L07	Commercial	KOR-C/M
218	Commercial	N_L08	Commercial	KOR-C/M
219	Commercial	N_L09	Commercial	KOR-C/M
220	Commercial	N_L10	Commercial	KOR-C/M
221	Commercial	N_O01	Commercial	KOR-C/M
222	Commercial	N_O02	Commercial	KOR-C/M
223	Commercial	N_O03	Commercial	KOR-C/M
224	Commercial	N_O04	Commercial	KOR-C/M
225	Commercial	N_O05	Commercial	KOR-C/M
226	Commercial	N_O06	Commercial	KOR-C/M
227	Commercial	N_O07	Commercial	KOR-C/M
228	Commercial	N_O08	Commercial	KOR-C/M
229	Commercial	N_O09	Commercial	KOR-C/M
230	Commercial	N_O10	Commercial	KOR-C/M
231	Commercial	N_W01	Commercial	KOR-C/M
232	Commercial	N_W02	Commercial	KOR-C/M
233	Commercial	N_W03	Commercial	KOR-C/M
234	Commercial	N_W04	Commercial	KOR-C/M
235	Commercial	N_W05	Commercial	KOR-C/M
236	Commercial	N_W06	Commercial	KOR-C/M
237	Commercial	N_W07	Commercial	KOR-C/M
238	Commercial	N_W08	Commercial	KOR-C/M
239	Commercial	N_W09	Commercial	KOR-C/M
240	Commercial	N_W10	Commercial	KOR-C/M
241	Silkie	G.S09	Silkie Brisson	KOR-KS
242	Silkie	G.S10	Silkie Brisson	KOR-KS
243	Silkie	G.S11	Silkie Brisson	KOR-KS

No.	Varieties	Sample code	Population	Population Code
244	Silkie	G.S12	Silkie Brisson	KOR-KS
245	Silkie	G.S13	Silkie Brisson	KOR-KS
246	Silkie	G.S14	Silkie Brisson	KOR-KS
247	Silkie	G.S15	Silkie Brisson	KOR-KS
248	Silkie	G.S16	Silkie Brisson	KOR-KS
249	Silkie	G.S17	Silkie Brisson	KOR-KS
250	Silkie	G.S18	Silkie Brisson	KOR-KS
251	Silkie	G.S19	Silkie Brisson	KOR-KS
252	Silkie	G.S20	Silkie Brisson	KOR-KS
253	Silkie	G.S21	Silkie Brisson	KOR-KS
254	Silkie	G.S22	Silkie Brisson	KOR-KS
255	Silkie	G.S23	Silkie Brisson	KOR-KS
256	Silkie	G.S24	Silkie Brisson	KOR-KS
257	Silkie	G.S25	Silkie Brisson	KOR-KS
258	Gray Brown	K.G.B19	Korean traditional chicken (Gray Brown)	KOR-KGB
259	Gray Brown	K.G.B20	Korean traditional chicken (Gray Brown)	KOR-KGB
260	Gray Brown	K.G.B21	Korean traditional chicken (Gray Brown)	KOR-KGB
261	Gray Brown	K.G.B22	Korean traditional chicken (Gray Brown)	KOR-KGB
262	Gray Brown	K.G.B23	Korean traditional chicken (Gray Brown)	KOR-KGB
263	Gray Brown	K.G.B24	Korean traditional chicken (Gray Brown)	KOR-KGB
264	Gray Brown	K.G.B25	Korean traditional chicken (Gray Brown)	KOR-KGB
265	Gray Brown	K.G.B26	Korean traditional chicken (Gray Brown)	KOR-KGB
266	Gray Brown	K.G.B27	Korean traditional chicken (Gray Brown)	KOR-KGB
267	Gray Brown	K.G.B28	Korean traditional chicken (Gray Brown)	KOR-KGB
268	Gray Brown	K.G.B29	Korean traditional chicken (Gray Brown)	KOR-KGB
269	Gray Brown	K.G.B30	Korean traditional chicken (Gray Brown)	KOR-KGB
270	Gray Brown	K.G.B31	Korean traditional chicken (Gray Brown)	KOR-KGB
271	Gray Brown	K.G.B32	Korean traditional chicken (Gray Brown)	KOR-KGB
272	Gray Brown	K.G.B33	Korean traditional chicken (Gray Brown)	KOR-KGB
273	Gray Brown	K.G.B34	Korean traditional chicken (Gray Brown)	KOR-KGB
274	Gray Brown	K.G.B35	Korean traditional chicken (Gray Brown)	KOR-KGB
275	Gray Brown	K.G.B36	Korean traditional chicken (Gray Brown)	KOR-KGB
276	Gray Brown	K.G.B37	Korean traditional chicken (Gray Brown)	KOR-KGB
277	Gray Brown	K.G.B38	Korean traditional chicken (Gray Brown)	KOR-KGB
278	Gray Brown	K.G.B39	Korean traditional chicken (Gray Brown)	KOR-KGB
279	Gray Brown	K.G.B40	Korean traditional chicken (Gray Brown)	KOR-KGB
280	Gray Brown	K.G.B41	Korean traditional chicken (Gray Brown)	KOR-KGB
281	Gray Brown	K.G.B42	Korean traditional chicken (Gray Brown)	KOR-KGB
282	Gray Brown	K.G.B43	Korean traditional chicken (Gray Brown)	KOR-KGB
283	Yellow Brown	KYB04	Korean traditional chicken (Yellow Brown)	KOR-KYB
284	Yellow Brown	KYB05	Korean traditional chicken (Yellow Brown)	KOR-KYB

No.	Varieties	Sample code	Population	Population Code
285	Yellow Brown	KYB06	Korean traditional chicken (Yellow Brown)	KOR-KYB
286	Yellow Brown	KYB07	Korean traditional chicken (Yellow Brown)	KOR-KYB
287	Yellow Brown	KYB08	Korean traditional chicken (Yellow Brown)	KOR-KYB
288	Yellow Brown	KYB09	Korean traditional chicken (Yellow Brown)	KOR-KYB
289	Yellow Brown	KYB10	Korean traditional chicken (Yellow Brown)	KOR-KYB
290	Yellow Brown	KYB11	Korean traditional chicken (Yellow Brown)	KOR-KYB
291	Yellow Brown	KYB12	Korean traditional chicken (Yellow Brown)	KOR-KYB
292	Yellow Brown	KYB13	Korean traditional chicken (Yellow Brown)	KOR-KYB
293	Yellow Brown	KYB14	Korean traditional chicken (Yellow Brown)	KOR-KYB
294	Yellow Brown	KYB15	Korean traditional chicken (Yellow Brown)	KOR-KYB
295	Yellow Brown	KYB16	Korean traditional chicken (Yellow Brown)	KOR-KYB
296	Yellow Brown	KYB17	Korean traditional chicken (Yellow Brown)	KOR-KYB
297	Yellow Brown	KYB18	Korean traditional chicken (Yellow Brown)	KOR-KYB
298	Yellow Brown	KYB19	Korean traditional chicken (Yellow Brown)	KOR-KYB
299	Yellow Brown	KYB20	Korean traditional chicken (Yellow Brown)	KOR-KYB
300	Leghorn	LH11	Leghorn (LH)	KOR-LH
301	Leghorn	LH12	Leghorn (LH)	KOR-LH
302	Leghorn	LH13	Leghorn (LH)	KOR-LH
303	Leghorn	LH14	Leghorn (LH)	KOR-LH
304	Leghorn	LH15	Leghorn (LH)	KOR-LH
305	Leghorn	LH16	Leghorn (LH)	KOR-LH
306	Leghorn	LH17	Leghorn (LH)	KOR-LH
307	Leghorn	LH18	Leghorn (LH)	KOR-LH
308	Leghorn	LH19	Leghorn (LH)	KOR-LH
309	Leghorn	LH20	Leghorn (LH)	KOR-LH
310	Leghorn	LH21	Leghorn (LH)	KOR-LH
311	Leghorn	LH22	Leghorn (LH)	KOR-LH
312	Leghorn	LH23	Leghorn (LH)	KOR-LH
313	Leghorn	LH24	Leghorn (LH)	KOR-LH
314	Leghorn	LH25	Leghorn (LH)	KOR-LH
315	Leghorn	LH26	Leghorn (LH)	KOR-LH
