# Supplementary material for: Divergent ancestry of Korean native and Thai chickens with independent gene pool retention by Korean commercial chickens
Source: Anim Biosci. 2025 Oct 22;39(3):250315. doi: 10.5713/ab.25.0315 (PMC12963744; doi:10.5713/ab.25.0315)
Supplement: Supplementary file 2 [file ab-25-0315-Supplementary-2.pdf]

Supplement 2. Genetic diversity of 315 individuals of Korean chicken varieties based on 28 microsatellite loci

Populations	Locus	N^1	$N_a{}^2$	AR^3	$N_{ea}{}^4$	I^5	$H_o{}^6$	$H_e{}^7$	M-ratio[8]	PIC^9	F^{10}	HWE^{11}
KOR-C/M	MCW0111	234	9.000	8.850	3.281	1.370	0.774	0.695	0.375	0.639	–0.113	***
	LEI0234	236	38.000	37.075	16.364	3.094	0.619	0.939	0.311	0.936	0.341	***
	MCW0206	235	12.000	11.845	4.501	1.746	0.796	0.778	0.600	0.746	–0.023	**
	MCW0016	229	10.000	9.773	5.195	1.786	0.952	0.808	0.385	0.780	–0.179	***
	MCW0103	227	7.000	6.894	2.834	1.266	0.559	0.647	0.069	0.593	0.135	***
	MCW0014	233	11.000	10.869	4.804	1.795	0.751	0.792	0.550	0.763	0.051	***
	ADL0268	236	16.000	15.800	7.109	2.178	0.831	0.859	0.139	0.844	0.034	***
	MCW0034	206	29.000	28.912	7.648	2.537	0.495	0.869	0.659	0.860	0.430	***
	LEI0166	228	14.000	13.439	3.401	1.523	0.618	0.706	0.117	0.661	0.124	***
	MCW0037	237	6.000	5.816	2.567	1.065	0.574	0.610	0.750	0.545	0.060	***
	MCW0295	227	15.000	14.786	5.756	2.011	0.665	0.826	0.536	0.806	0.195	***
	LEI0094	222	18.000	17.800	6.193	2.112	0.842	0.839	0.500	0.820	–0.005	***
	MCW0216	233	23.000	22.227	8.171	2.457	0.532	0.878	0.605	0.867	0.394	***
	MCW0222	231	11.000	10.879	5.517	1.934	0.429	0.819	0.500	0.796	0.477	***
	MCW0098	231	11.000	10.622	3.871	1.587	0.567	0.742	0.550	0.704	0.235	***
	MCW0078	203	15.000	15.000	7.808	2.290	0.468	0.872	0.136	0.860	0.463	***
	MCW0081	233	17.000	16.452	4.517	1.859	0.455	0.779	0.080	0.750	0.416	***
	MCW0183	228	26.000	25.207	4.268	2.093	0.368	0.766	0.419	0.750	0.519	***
	MCW0067	226	10.000	9.988	3.980	1.580	0.274	0.749	0.357	0.709	0.634	***
	MCW0248	229	6.000	5.986	2.041	0.950	0.480	0.510	0.600	0.451	0.058	***
	LEI0192	240	27.000	26.434	5.845	2.308	0.496	0.829	0.142	0.813	0.402	***
	ADL0112	228	14.000	13.670	6.150	2.090	0.658	0.837	0.538	0.821	0.214	***
	MCW0165	240	6.000	5.999	3.582	1.406	0.613	0.721	0.429	0.673	0.150	***
	ADL0278	232	14.000	13.859	4.321	1.933	0.353	0.769	0.500	0.751	0.540	***
	MCW0104	223	24.000	23.863	9.391	2.571	0.592	0.894	0.103	0.885	0.338	***
	MCW0123	237	7.000	6.980	4.001	1.554	0.717	0.750	0.233	0.711	0.044	***
	MCW0330	208	30.000	29.903	9.019	2.610	0.673	0.889	0.545	0.880	0.243	***
	MCW0069	219	16.000	15.927	9.913	2.458	0.594	0.899	0.571	0.891	0.340	***
	Mean	228.250	15.786	15.531	5.787	1.934	0.598	0.788	0.250	0.761	0.233	
	SE	1.793	1.575	1.546	0.560	0.096	0.030	0.018	0.132	0.021	0.040	

Populations	Locus	N^1	$N_a{}^2$	AR^3	$N_{ea}{}^4$	I^5	$H_o{}^6$	$H_e{}^7$	M-ratio[8]	PIC^9	F^{10}	HWE^{11}
KOR-KS	MCW0111	17	4.000	3.316	2.379	0.986	0.882	0.580	0.500	0.490	–0.522	ns
	LEI0234	17	17.000	11.842	12.042	2.647	0.882	0.917	0.118	0.911	0.038	*
	MCW0206	17	4.000	3.316	2.267	0.963	0.882	0.559	0.667	0.477	–0.579	ns
	MCW0016	17	4.000	3.921	2.876	1.202	0.941	0.652	0.667	0.599	–0.443	ns
	MCW0103	17	2.000	2.000	1.895	0.665	0.647	0.472	0.500	0.361	–0.370	ns
	MCW0014	17	6.000	5.476	4.857	1.653	0.941	0.794	0.400	0.763	–0.185	*
	ADL0268	15	5.000	4.194	2.961	1.242	1.000	0.662	0.278	0.604	–0.510	***
	MCW0034	14	8.000	7.352	6.533	1.959	1.000	0.847	0.286	0.828	–0.181	*
	LEI0166	10	6.000	5.795	4.000	1.543	1.000	0.750	0.500	0.711	–0.333	*
	MCW0037	16	3.000	2.996	2.296	0.956	0.750	0.564	0.750	0.503	–0.329	ns
	MCW0295	9	3.000	3.000	2.160	0.854	0.889	0.537	0.500	0.441	–0.655	ns
	LEI0094	12	8.000	7.281	4.299	1.757	0.750	0.767	0.222	0.744	0.023	***
	MCW0216	17	7.000	5.895	3.828	1.598	0.824	0.739	0.292	0.710	–0.115	**
	MCW0222	17	3.000	2.992	2.198	0.929	0.765	0.545	0.750	0.486	–0.403	ns
	MCW0098	17	4.000	3.929	2.481	1.128	0.412	0.597	0.333	0.555	0.310	ns
	MCW0078	17	3.000	2.961	2.481	0.974	0.647	0.597	0.750	0.510	–0.084	ns
	MCW0081	17	2.000	1.994	1.410	0.466	0.353	0.291	0.100	0.248	–0.214	ns
	MCW0183	17	4.000	3.529	2.979	1.168	0.647	0.664	0.074	0.599	0.026	ns
	MCW0067	17	4.000	3.436	2.418	1.022	0.706	0.587	0.667	0.510	–0.204	ns
	MCW0248	17	5.000	4.479	3.026	1.280	1.000	0.670	0.500	0.611	–0.494	ns
	LEI0192	17	5.000	4.599	2.949	1.293	0.706	0.661	0.104	0.613	–0.068	ns
	ADL0112	17	3.000	2.529	2.102	0.801	0.941	0.524	0.750	0.414	–0.795	**
	MCW0165	17	4.000	3.474	1.871	0.883	0.235	0.465	0.333	0.429	0.494	**
	ADL0278	17	1.000	1.000	1.000	0.000	0.000	0.000	0.214	0.000	0.000	ns
	MCW0104	11	3.000	2.974	2.283	0.916	0.727	0.562	0.750	0.476	–0.294	ns
	MCW0123	17	2.000	2.000	2.000	0.693	1.000	0.500	0.083	0.375	–1.000	***
	MCW0330	17	3.000	2.786	2.240	0.876	0.941	0.554	0.107	0.452	–0.700	***
	MCW0069	17	2.000	1.529	1.061	0.133	0.059	0.057	0.167	0.055	–0.030	ns
	Mean	15.857	4.464	3.950	3.032	1.092	0.733	0.575	0.224	0.517	–0.282	
	SE	0.448	0.572	0.416	0.399	0.101	0.053	0.038	0.107	0.038	0.062	

Populations	Locus	N[1]	N_a[2]	AR[3]	N_{ea}[4]	I[5]	H_o[6]	H_e[7]	M-ratio[8]	PIC[9]	F[10]	HWE[11]
KOR-KGB	MCW0111	19	6.000	6.000	2.865	1.307	0.737	0.651	0.375	0.608	–0.132	ns
	LEI0234	24	12.000	10.872	6.295	2.073	0.833	0.841	0.133	0.823	0.009	***
	MCW0206	25	7.000	6.650	3.655	1.528	0.920	0.726	0.500	0.688	–0.267	***
	MCW0016	20	6.000	5.999	4.848	1.663	0.950	0.794	0.500	0.763	–0.197	*
	MCW0103	22	3.000	3.000	2.822	1.066	0.864	0.646	0.750	0.571	–0.338	***
	MCW0014	22	7.000	6.726	4.033	1.587	1.000	0.752	0.500	0.717	–0.330	ns
	ADL0268	25	10.000	8.692	4.296	1.706	1.000	0.767	0.833	0.731	–0.303	ns
	MCW0034	25	8.000	7.887	5.102	1.829	0.760	0.804	0.286	0.780	0.055	***
	LEI0166	24	4.000	3.792	2.887	1.135	0.667	0.654	0.222	0.585	–0.020	ns
	MCW0037	25	5.000	4.760	3.613	1.373	0.800	0.723	0.625	0.673	–0.106	ns
	MCW0295	24	7.000	6.543	3.512	1.506	0.750	0.715	0.500	0.680	–0.049	ns
	LEI0094	25	9.000	8.464	6.068	1.941	0.840	0.835	0.237	0.815	–0.006	*
	MCW0216	25	10.000	9.166	4.630	1.824	0.840	0.784	0.357	0.756	–0.071	ns
	MCW0222	25	3.000	3.000	2.273	0.950	0.480	0.560	0.750	0.499	0.143	**
	MCW0098	25	5.000	4.760	3.289	1.319	0.680	0.696	0.357	0.642	0.023	ns
	MCW0078	25	5.000	4.989	3.360	1.389	0.640	0.702	0.313	0.662	0.089	***
	MCW0081	25	3.000	2.760	1.541	0.594	0.360	0.351	0.150	0.302	–0.025	ns
	MCW0183	25	6.000	5.455	1.997	1.025	0.440	0.499	0.150	0.463	0.119	**
	MCW0067	24	6.000	5.959	3.932	1.559	0.500	0.746	0.500	0.713	0.329	*
	MCW0248	22	6.000	5.711	2.933	1.298	0.909	0.659	0.600	0.610	–0.379	***
	LEI0192	23	9.000	8.443	3.875	1.670	0.348	0.742	0.052	0.709	0.531	***
	ADL0112	24	4.000	4.000	3.088	1.230	1.000	0.676	0.667	0.616	–0.479	***
	MCW0165	24	3.000	3.000	2.228	0.932	0.292	0.551	0.750	0.487	0.471	***
	ADL0278	25	2.000	1.760	1.041	0.098	0.040	0.039	0.080	0.038	–0.020	ns
	MCW0104	25	2.000	2.000	2.000	0.693	1.000	0.500	0.333	0.375	–1.000	***
	MCW0123	25	3.000	3.000	2.599	1.020	1.000	0.615	0.125	0.541	–0.625	***
	MCW0330	25	2.000	2.000	2.000	0.693	1.000	0.500	0.200	0.375	–1.000	***
	MCW0069	25	7.000	6.637	2.073	1.171	0.480	0.518	0.583	0.499	0.073	ns
	Mean	24.000	5.714	5.429	3.316	1.292	0.719	0.645	0.238	0.597	–0.125	
	SE	0.304	0.509	0.459	0.243	0.085	0.049	0.032	0.080	0.033	0.068	

Populations	Locus	N^1	$N_a{}^2$	AR^3	$N_{ea}{}^4$	I^5	$H_o{}^6$	$H_e{}^7$	M-ratio[8]	PIC^9	F^{10}	HWE^{11}
KOR-KYB	MCW0111	14	4.000	4.000	3.769	1.358	0.929	0.735	0.500	0.687	–0.264	ns
	LEI0234	17	11.000	9.887	3.828	1.814	0.412	0.739	0.076	0.720	0.443	***
	MCW0206	17	8.000	7.620	5.558	1.852	1.000	0.820	0.063	0.797	–0.219	***
	MCW0016	14	6.000	6.000	4.170	1.546	1.000	0.760	0.500	0.721	–0.315	ns
	MCW0103	17	3.000	3.000	2.934	1.087	0.882	0.659	0.750	0.585	–0.339	ns
	MCW0014	17	7.000	6.816	5.161	1.766	1.000	0.806	0.500	0.780	–0.240	ns
	ADL0268	16	3.000	3.000	2.738	1.051	1.000	0.635	0.750	0.561	–0.575	**
	MCW0034	16	6.000	5.749	3.507	1.447	0.438	0.715	0.333	0.674	0.388	*
	LEI0166	17	4.000	3.647	1.973	0.849	0.647	0.493	0.500	0.419	–0.312	***
	MCW0037	17	3.000	2.973	2.206	0.869	0.588	0.547	0.750	0.449	–0.076	ns
	MCW0295	17	6.000	5.640	2.223	1.169	0.706	0.550	0.429	0.523	–0.283	ns
	LEI0094	17	10.000	9.616	8.377	2.189	0.706	0.881	0.313	0.868	0.198	ns
	MCW0216	17	8.000	7.267	2.820	1.440	0.647	0.645	0.286	0.618	–0.003	ns
	MCW0222	17	4.000	3.973	3.042	1.206	0.588	0.671	0.667	0.608	0.124	***
	MCW0098	17	3.000	3.000	2.198	0.929	0.765	0.545	0.750	0.486	–0.403	ns
	MCW0078	16	4.000	3.999	3.103	1.237	0.563	0.678	0.667	0.621	0.170	ns
	MCW0081	17	5.000	4.647	2.580	1.164	0.765	0.612	0.208	0.563	–0.249	ns
	MCW0183	17	7.000	6.646	4.983	1.721	0.882	0.799	0.130	0.770	–0.104	*
	MCW0067	17	5.000	4.973	4.158	1.495	0.647	0.760	0.417	0.719	0.148	**
	MCW0248	17	5.000	4.647	2.676	1.178	0.882	0.626	0.500	0.571	–0.409	ns
	LEI0192	15	7.000	6.867	4.639	1.692	0.600	0.784	0.045	0.754	0.235	ns
	ADL0112	16	3.000	3.000	2.723	1.043	1.000	0.633	0.500	0.556	–0.580	*
	MCW0165	16	4.000	3.875	2.876	1.153	0.875	0.652	0.400	0.588	–0.341	ns
	ADL0278	17	3.000	4.647	3.167	1.078	0.763	0.675	0.167	0.723	–0.267	ns
	MCW0104	17	5.000	4.620	1.979	0.964	0.471	0.495	0.167	0.451	0.049	***
	MCW0123	17	2.000	2.000	2.000	0.693	1.000	0.500	0.100	0.375	–1.000	***
	MCW0330	17	3.000	3.000	2.701	1.037	0.824	0.630	0.075	0.551	–0.308	***
	MCW0069	17	5.000	4.970	3.026	1.320	1.000	0.670	0.625	0.626	–0.494	ns
	Mean	16.536	5.071	5.003	3.320	1.260	0.743	0.644	0.176	0.594	–0.176	
	SE	0.167	0.442	0.379	0.276	0.082	0.045	0.031	0.053	0.032	0.062	

Populations	Locus	N[1]	N_a[2]	AR[3]	N_{ea}[4]	I[5]	H_o[6]	H_e[7]	M-ratio[8]	PIC[9]	F[10]	HWE[11]
KOR-LH	MCW0111	16	3.000	2.938	2.107	0.806	0.938	0.525	0.375	0.416	–0.784	**
	LEI0234	16	14.000	13.617	9.481	2.429	0.938	0.895	0.119	0.886	–0.048	ns
	MCW0206	16	10.000	9.802	4.923	1.905	1.000	0.797	0.417	0.774	–0.255	*
	MCW0016	16	4.000	3.938	2.681	1.096	1.000	0.627	0.500	0.552	–0.595	**
	MCW0103	16	2.000	2.000	2.000	0.693	1.000	0.500	1.000	0.375	–1.000	***
	MCW0014	16	8.000	7.813	4.876	1.778	1.000	0.795	0.571	0.769	–0.258	*
	ADL0268	16	4.000	3.998	2.709	1.123	0.938	0.631	0.667	0.559	–0.486	***
	MCW0034	16	6.000	5.994	3.556	1.488	0.563	0.719	0.214	0.680	0.217	***
	LEI0166	16	6.000	5.750	2.073	1.042	0.500	0.518	0.353	0.473	0.034	**
	MCW0037	16	3.000	2.998	2.246	0.882	0.813	0.555	0.750	0.456	–0.465	ns
	MCW0295	16	7.000	6.748	1.875	1.066	0.563	0.467	0.500	0.449	–0.205	ns
	LEI0094	16	4.000	3.935	2.404	0.998	0.813	0.584	0.250	0.496	–0.391	***
	MCW0216	16	6.000	5.938	4.830	1.650	0.750	0.793	0.250	0.761	0.054	***
	MCW0222	16	3.000	3.000	1.992	0.845	0.625	0.498	0.750	0.436	–0.255	ns
	MCW0098	16	3.000	3.000	2.381	0.939	0.813	0.580	0.750	0.489	–0.401	ns
	MCW0078	16	4.000	4.000	2.829	1.185	0.563	0.646	0.667	0.591	0.130	ns
	MCW0081	16	5.000	4.998	3.683	1.436	0.563	0.729	0.192	0.687	0.228	ns
	MCW0183	16	7.000	6.810	2.753	1.372	0.438	0.637	0.130	0.606	0.313	***
	MCW0067	16	7.000	6.933	4.655	1.704	0.250	0.785	0.583	0.756	0.682	***
	MCW0248	16	2.000	2.000	1.600	0.562	0.500	0.375	1.000	0.305	–0.333	ns
	LEI0192	15	5.000	5.000	3.333	1.345	0.267	0.700	0.114	0.648	0.619	***
	ADL0112	16	2.000	2.000	2.000	0.693	1.000	0.500	1.000	0.375	–1.000	***
	MCW0165	16	3.000	2.998	2.107	0.850	0.813	0.525	0.300	0.438	–0.546	ns
	ADL0278	16	4.000	3.935	1.484	0.672	0.063	0.326	0.222	0.308	0.808	***
	MCW0104	16	8.000	7.748	2.599	1.398	0.375	0.615	0.286	0.592	0.390	***
	MCW0123	16	3.000	3.000	2.359	0.934	1.000	0.576	0.150	0.486	–0.736	**
	MCW0330	16	3.000	3.000	1.476	0.613	0.188	0.322	0.115	0.299	0.418	***
	MCW0069	16	9.000	8.810	6.024	1.955	0.875	0.834	0.563	0.814	–0.049	ns
	Mean	15.964	5.179	5.096	3.108	1.195	0.684	0.609	0.261	0.553	–0.140	
	SE	0.036	0.532	0.515	0.326	0.088	0.054	0.028	0.125	0.031	0.092	

[1]Number of samples (N); [2]number of alleles (N_a); [3]allelic richness (AR); [4]number of effective alleles (N_{ea}); [5]Shannon's information index (I); [6]observed heterozygosity (H_o); [7]expected heterozygosity (H_e); [8]M-ratio; [9]polymorphic information content (PIC); [10]fixation index (F); [11]*HWE* (Hardy-Weinberg Equilibrium); not significant (ns); $p<0.05$ (*); $p<0.01$ (**); $p<0.001$ (***); KOR-C/M = Korean commercial chicken; KOR-KS = Silkie; KOR-KGB = Korean traditional chicken (Gray Brown); KOR-KYB = Korean traditional chicken (Yellow Brown); KOR-LH = Leghorn (LH).
