# Supplementary material for: Divergent ancestry of Korean native and Thai chickens with independent gene pool retention by Korean commercial chickens
Source: Anim Biosci. 2025 Oct 22;39(3):250315. doi: 10.5713/ab.25.0315 (PMC12963744; doi:10.5713/ab.25.0315)
Supplement: Supplementary file 3 [file ab-25-0315-Supplementary-3.pdf]

Supplement 3. Observed and expected heterozygosity of Korean chicken varieties based on 28 microsatellite loci.

Breeds	H_o	H_e	df	t-test	*p*-value
KOR–C/M	0.598±0.030	0.788±0.018	–0.19	–5.431	0.000
KOR–KS	0.733±0.053	0.575±0.038	0.158	2.423	0.022
KOR–KGB	0.719±0.049	0.645±0.032	0.074	1.264	0.213
KOR–KYB	0.743±0.045	0.644±0.031	0.099	1.812	0.081
KOR–LH	0.684±0.054	0.609±0.028	0.075	1.233	0.230

df = difference of means; KOR-C/M = Korean commercial chicken; KOR-KS = Silkie; KOR-KGB = Korean traditional chicken (Gray Brown); KOR-KYB = Korean traditional chicken (Yellow Brown); KOR-LH = Leghorn (LH)
