# Supplementary material for: Divergent ancestry of Korean native and Thai chickens with independent gene pool retention by Korean commercial chickens
Source: Anim Biosci. 2025 Oct 22;39(3):250315. doi: 10.5713/ab.25.0315 (PMC12963744; doi:10.5713/ab.25.0315)
Supplement: Supplementary file 4 [file ab-25-0315-Supplementary-4.pdf]

Supplement 4. Comparison of genetic diversity parameters between Korean chicken varieties based on 28 microsatellite loci.

Heterozygosity	Population 1	Population 2	df	SE	t-test	*p*–value
	KOR-CM	KOR-KS	–0.135	0.061	–2.217	0.035
	KOR-CM	KOR-KGB	–0.121	0.057	–2.106	0.041
	KOR-CM	KOR-KYB	–0.145	0.054	–2.681	0.011
	KOR-CM	KOR-LH	–0.086	0.062	–1.392	0.176
	KOR-KS	KOR-KGB	0.014	0.072	0.194	0.847
H_o	KOR-KS	KOR-KYB	–0.010	0.070	–0.144	0.887
	KOR-KS	KOR-LH	0.049	0.076	0.648	0.522
	KOR-KGB	KOR-KYB	–0.024	0.067	–0.361	0.720
	KOR-KGB	KOR-LH	0.035	0.073	0.480	0.634
	KOR-KYB	KOR-LH	0.059	0.070	0.839	0.408
	KOR-CM	KOR-KS	0.213	0.042	5.066	0.000
	KOR-CM	KOR-KGB	0.143	0.037	3.895	0.000
	KOR-CM	KOR-KYB	0.144	0.036	4.017	0.000
	KOR-CM	KOR-LH	0.179	0.033	5.378	0.000
	KOR-KS	KOR-KGB	–0.070	0.050	–1.409	0.168
H_e	KOR-KS	KOR-KYB	–0.069	0.049	–1.407	0.169
	KOR-KS	KOR-LH	–0.034	0.047	–0.720	0.477
	KOR-KGB	KOR-KYB	0.001	0.045	0.022	0.982
	KOR-KGB	KOR-LH	0.036	0.043	0.847	0.402
	KOR-KYB	KOR-LH	0.035	0.042	0.838	0.409

df = difference of means; SE = Standard error; KOR-C/M = Korean commercial chicken; KOR-KS = Silkie; KOR-KGB = Korean traditional chicken (Gray Brown); KOR-KYB = Korean traditional chicken (Yellow Brown); KOR-LH = Leghorn (LH)
