# Supplementary material for: Divergent ancestry of Korean native and Thai chickens with independent gene pool retention by Korean commercial chickens
Source: Anim Biosci. 2025 Oct 22;39(3):250315. doi: 10.5713/ab.25.0315 (PMC12963744; doi:10.5713/ab.25.0315)
Supplement: Supplementary file 5 [file ab-25-0315-Supplementary-5.pdf]

Supplement 5. Average F_{IS} and relatedness (r) among five varieties of Korean chicken.

Varieties	F_{IS}	Relatedness
KOR-C/M	0.226	–0.003
KOR-KS	–0.262	–0.022
KOR-KGB	–0.161	–0.030
KOR-KYB	–0.0191	–0.030
KOR-LH	–0.169	–0.034

KOR-C/M = Korean commercial chicken; KOR-KS = Silkie; KOR-KGB = Korean traditional chicken (Gray Brown); KOR-KYB = Korean traditional chicken (Yellow Brown); KOR-LH = Leghorn (LH)
