# Supplementary material for: Divergent ancestry of Korean native and Thai chickens with independent gene pool retention by Korean commercial chickens
Source: Anim Biosci. 2025 Oct 22;39(3):250315. doi: 10.5713/ab.25.0315 (PMC12963744; doi:10.5713/ab.25.0315)
Supplement: Supplementary file 6 [file ab-25-0315-Supplementary-6.pdf]

Supplement 6. Distributions of genetic relatedness values (r) and inbreeding coefficients (F_{IS}) for the Korean chicken varieties.

Population 1	Population 2	Relatedness value (r)		Inbreeding coefficient (F_{IS})	
		Density	p-value	Density	p-value
KOR-C/M	KOR-KS	0.321	0.000	1.000	0.000
KOR-C/M	KOR-KGB	0.438	0.000	1.000	0.000
KOR-C/M	KOR-KYB	0.461	0.000	1.000	0.000
KOR-C/M	KOR-LH	0.425	0.000	1.000	0.000
KOR-C/M	KOR-KS	0.321	0.000	1.000	0.000
KOR-C/M	KOR-KGB	0.438	0.000	1.000	0.000
KOR-C/M	KOR-KYB	0.461	0.000	1.000	0.000
KOR-C/M	KOR-LH	0.425	0.000	1.000	0.000
KOR-C/M	KOR-KS	0.441	0.000	1.000	0.000
KOR-C/M	KOR-KGB	0.326	0.000	1.000	0.000
KOR-C/M	KOR-KYB	0.437	0.000	1.000	0.000
KOR-C/M	KOR-LH	0.451	0.000	1.000	0.000
KOR-C/M	KOR-KS	0.381	0.000	1.000	0.000
KOR-C/M	KOR-KGB	0.452	0.000	1.000	0.000
KOR-C/M	KOR-KYB	0.381	0.000	1.000	0.000
KOR-C/M	KOR-LH	0.455	0.000	1.000	0.000
KOR-C/M	Allpop	0.009	0.187	0.781	0.000
KOR-KS	KOR-KGB	0.134	0.070	0.784	0.000
KOR-KS	KOR-KYB	0.164	0.013	0.647	0.001
KOR-KS	KOR-LH	0.130	0.110	0.765	0.000
KOR-KS	Allpop	0.312	0.000	0.838	0.000
KOR-KGB	KOR-KYB	0.110	0.380	0.468	0.014
KOR-KGB	KOR-LH	0.079	0.816	0.213	0.720
KOR-KGB	Allpop	0.429	0.000	0.703	0.000
KOR-KYB	KOR-LH	0.086	0.730	0.314	0.327
KOR-KYB	Allpop	0.452	0.000	0.722	0.000
KOR-LH	Allpop	0.417	0.000	0.673	0.000

KOR-C/M = Korean commercial chicken; KOR-KS = Silkie; KOR-KGB = Korean traditional chicken (Gray Brown); KOR-KYB = Korean traditional chicken (Yellow Brown); KOR-LH = Leghorn (LH)
