# Supplementary material for: Divergent ancestry of Korean native and Thai chickens with independent gene pool retention by Korean commercial chickens
Source: Anim Biosci. 2025 Oct 22;39(3):250315. doi: 10.5713/ab.25.0315 (PMC12963744; doi:10.5713/ab.25.0315)
Supplement: Supplementary file 7 [file ab-25-0315-Supplementary-7.pdf]

Supplement 7. Pairwise comparison of genetic differentiation (F_{ST}), F_{ST}^{ENA} values with ENA correction for null alleles, and R_{ST} values between five Korean chicken varieties using FSTAT version 2.9.3 (Goudet, 1995).

Combination	F_{ST}	F_{ST}^{ENA}	R_{ST}
KOR-C/M x KOR-KS	0.141*	0.152	0.194
KOR-C/M x KOR-KGB	0.128*	0.133	0.241
KOR-C/M x KOR-KYB	0.131*	0.137	0.173
KOR-C/M x KOR-LH	0.123*	0.124	0.155
KOR-KS x KOR-KGB	0.105*	0.104	0.118
KOR-KS x KOR-KYB	0.182*	0.177	0.215
KOR-KS x KOR-LH	0.218*	0.218	0.352
KOR-KGB x KOR-KYB	0.108*	0.105	0.217
KOR-KGB x KOR-LH	0.169*	0.168	0.426
KOR-KYB x KOR-LH	0.094*	0.094	0.129

*p-value < 0.05; KOR-C/M = Korean commercial chicken; KOR-KS = Silkie; KOR-KGB = Korean traditional chicken (Gray Brown); KOR-KYB = Korean traditional chicken (Yellow Brown); KOR-LH = Leghorn (LH)

Goudet, J. (1995). FSTAT (version 2.9.3): A computer program to calculate F-statistics. *Journal of Heredity*, 86(6), 485-486.
