# Supplementary material for: Divergent ancestry of Korean native and Thai chickens with independent gene pool retention by Korean commercial chickens
Source: Anim Biosci. 2025 Oct 22;39(3):250315. doi: 10.5713/ab.25.0315 (PMC12963744; doi:10.5713/ab.25.0315)
Supplement: Supplementary file 8 [file ab-25-0315-Supplementary-8.pdf]

Supplement 8. Analysis of molecular variance (AMOVA) for Korean chicken varieties based on 28 microsatellite loci.

Source of variation	df	Sum of squares	Variance components	Percentage of variation
among populations	4	276.659	0.986	13.55
within individual	625	3932.613	6.292	86.45
Total	629	4209.271	7.287	100%

df = degree of freedom
