# Supplementary material for: Divergent ancestry of Korean native and Thai chickens with independent gene pool retention by Korean commercial chickens
Source: Anim Biosci. 2025 Oct 22;39(3):250315. doi: 10.5713/ab.25.0315 (PMC12963744; doi:10.5713/ab.25.0315)
Supplement: Supplementary file 9 [file ab-25-0315-Supplementary-9.pdf]

Supplement 9. Results of pairwise Nei's genetic distance (*D*) among Korean chicken varieties, based on 28 microsatellite loci**.**

Nei's D	KOR-C/M	KOR-KS	KOR-KGB	KOR-KYB	KOR-LH
KOR-C/M	0.000				
KOR-KS	0.526	0.000			
KOR-KGB	0.536	0.252	0.000		
KOR-KYB	0.593	0.514	0.304	0.000	
KOR-LH	0.490	0.619	0.492	0.258	0.000

KOR-C/M = Korean commercial chicken; KOR-KS = Silkie; KOR-KGB = Korean traditional chicken (Gray Brown); KOR-KYB = Korean traditional chicken (Yellow Brown); KOR-LH = Leghorn (LH)
