# Supplementary material for: Divergent ancestry of Korean native and Thai chickens with independent gene pool retention by Korean commercial chickens
Source: Anim Biosci. 2025 Oct 22;39(3):250315. doi: 10.5713/ab.25.0315 (PMC12963744; doi:10.5713/ab.25.0315)
Supplement: Supplementary file 10 [file ab-25-0315-Supplementary-10.pdf]

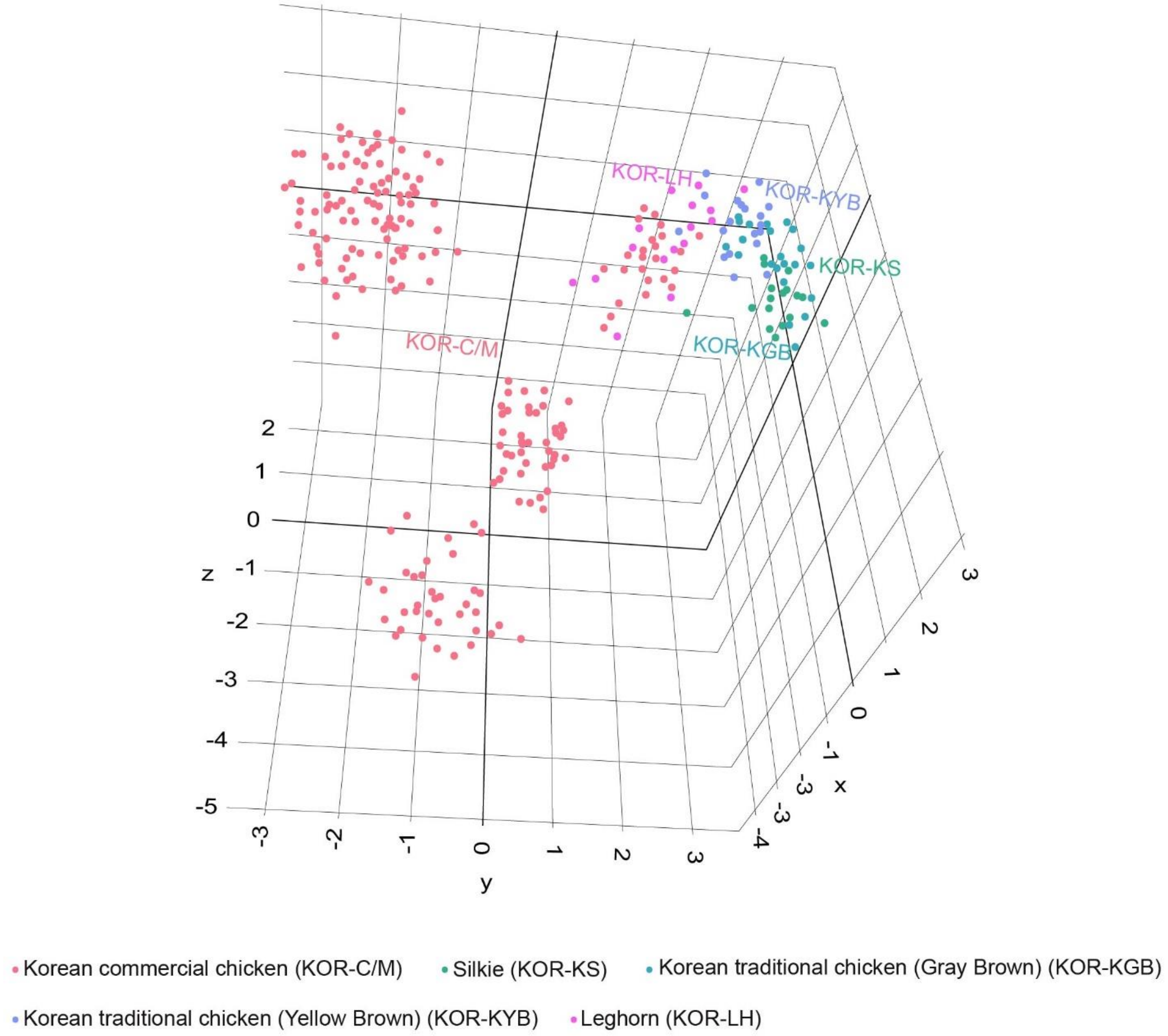

Supplement 10. Principal coordinate analysis (PCoA) of five Korean chickens. Dots represent different individuals and colours represent different genetic clusters
