# Supplementary material for: Divergent ancestry of Korean native and Thai chickens with independent gene pool retention by Korean commercial chickens
Source: Anim Biosci. 2025 Oct 22;39(3):250315. doi: 10.5713/ab.25.0315 (PMC12963744; doi:10.5713/ab.25.0315)
Supplement: Supplementary file 11 [file ab-25-0315-Supplementary-11.pdf]

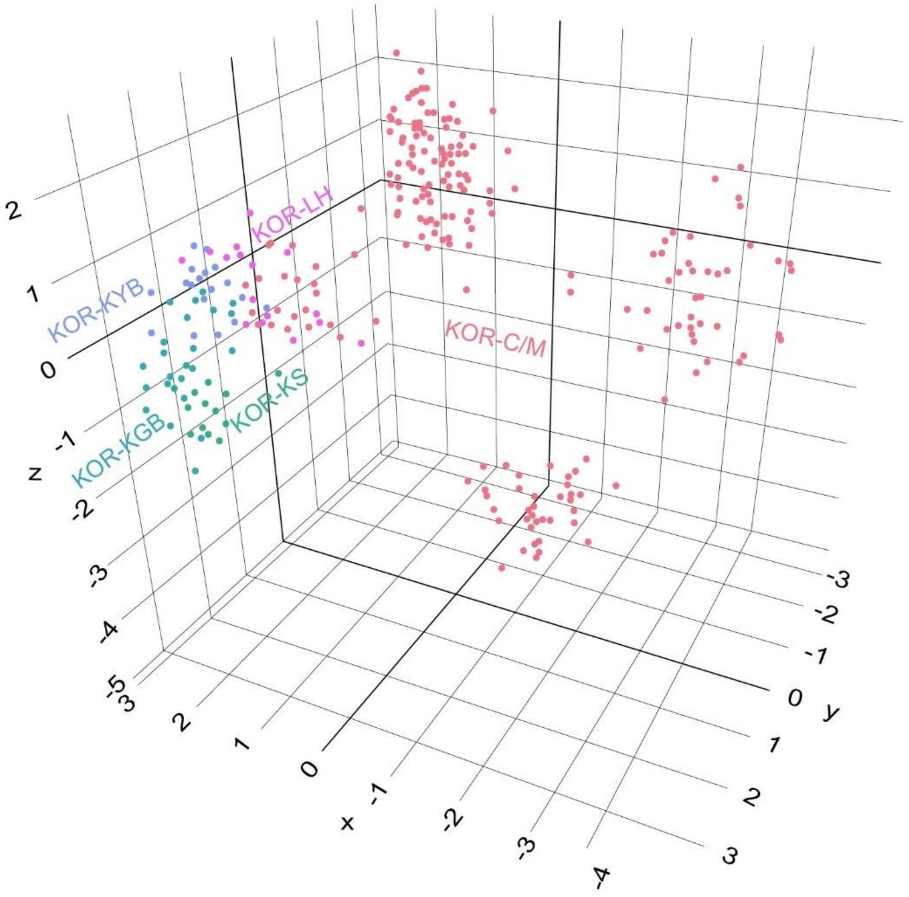

• Korean commercial chicken (KOR-C/M) • Silkie (KOR-KS) • Korean traditional chicken (Gray Brown) (KOR-KGB)

• Korean traditional chicken (Yellow Brown) (KOR-KYB) • Leghorn (KOR-LH)

Supplement 11. Discriminant Analysis of Principal Components (DAPC) of five Korean chicken varieties. Different colours indicate scatter plots based on DAPC output for assigned genetic clusters. Dots represent different individuals
