# Supplementary material for: Divergent ancestry of Korean native and Thai chickens with independent gene pool retention by Korean commercial chickens
Source: Anim Biosci. 2025 Oct 22;39(3):250315. doi: 10.5713/ab.25.0315 (PMC12963744; doi:10.5713/ab.25.0315)
Supplement: Supplementary file 13 [file ab-25-0315-Supplementary-13.pdf]

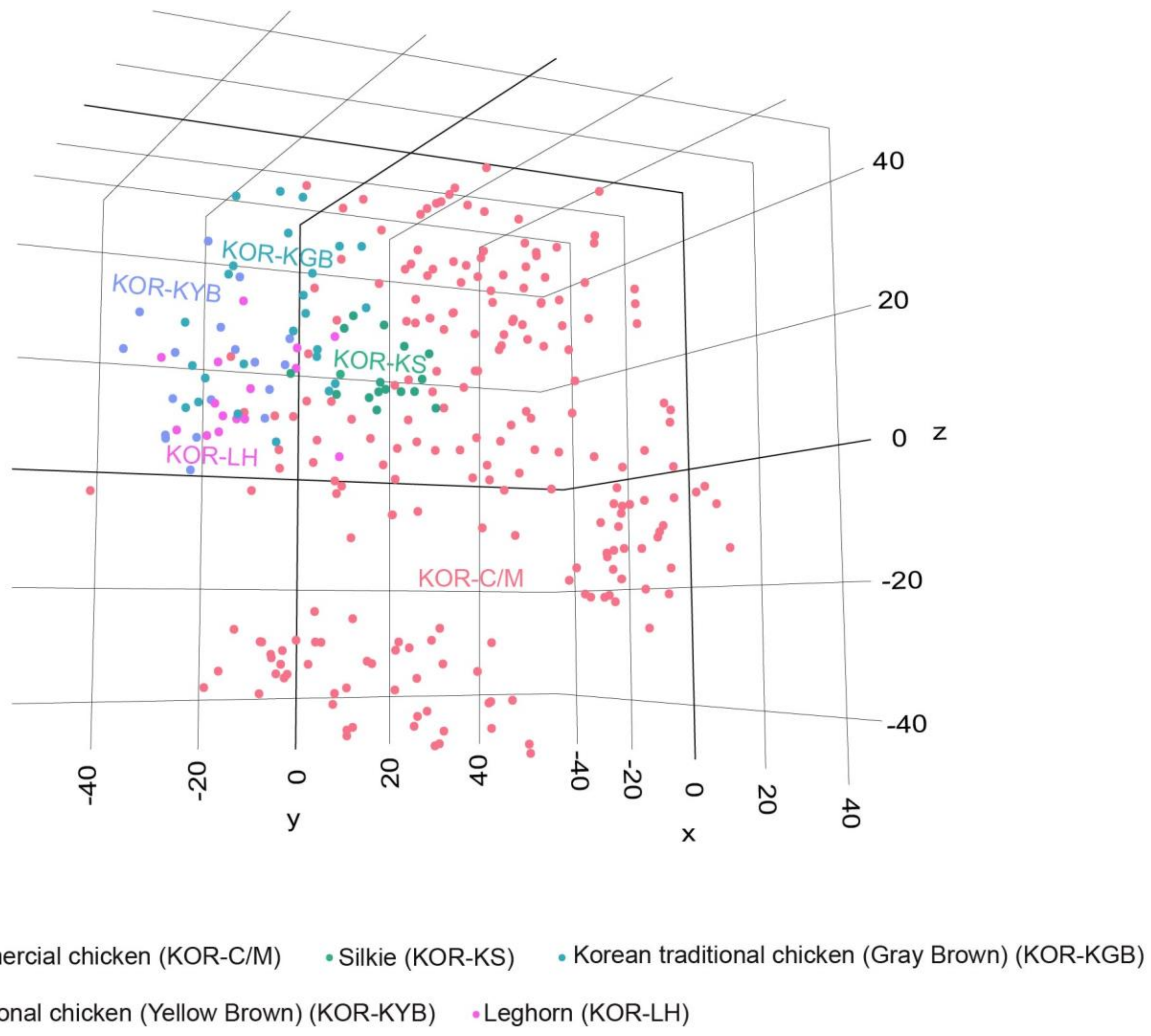

Supplement 13. Multidimensional Scaling (MDS) of five Korean chicken varieties. The scatter plot illustrates the genetic clusters based on the different colors. Dots represent different individuals and colours represent different genetic clusters
