# Supplementary material for: Divergent ancestry of Korean native and Thai chickens with independent gene pool retention by Korean commercial chickens
Source: Anim Biosci. 2025 Oct 22;39(3):250315. doi: 10.5713/ab.25.0315 (PMC12963744; doi:10.5713/ab.25.0315)
Supplement: Supplementary file 14 [file ab-25-0315-Supplementary-14.pdf]

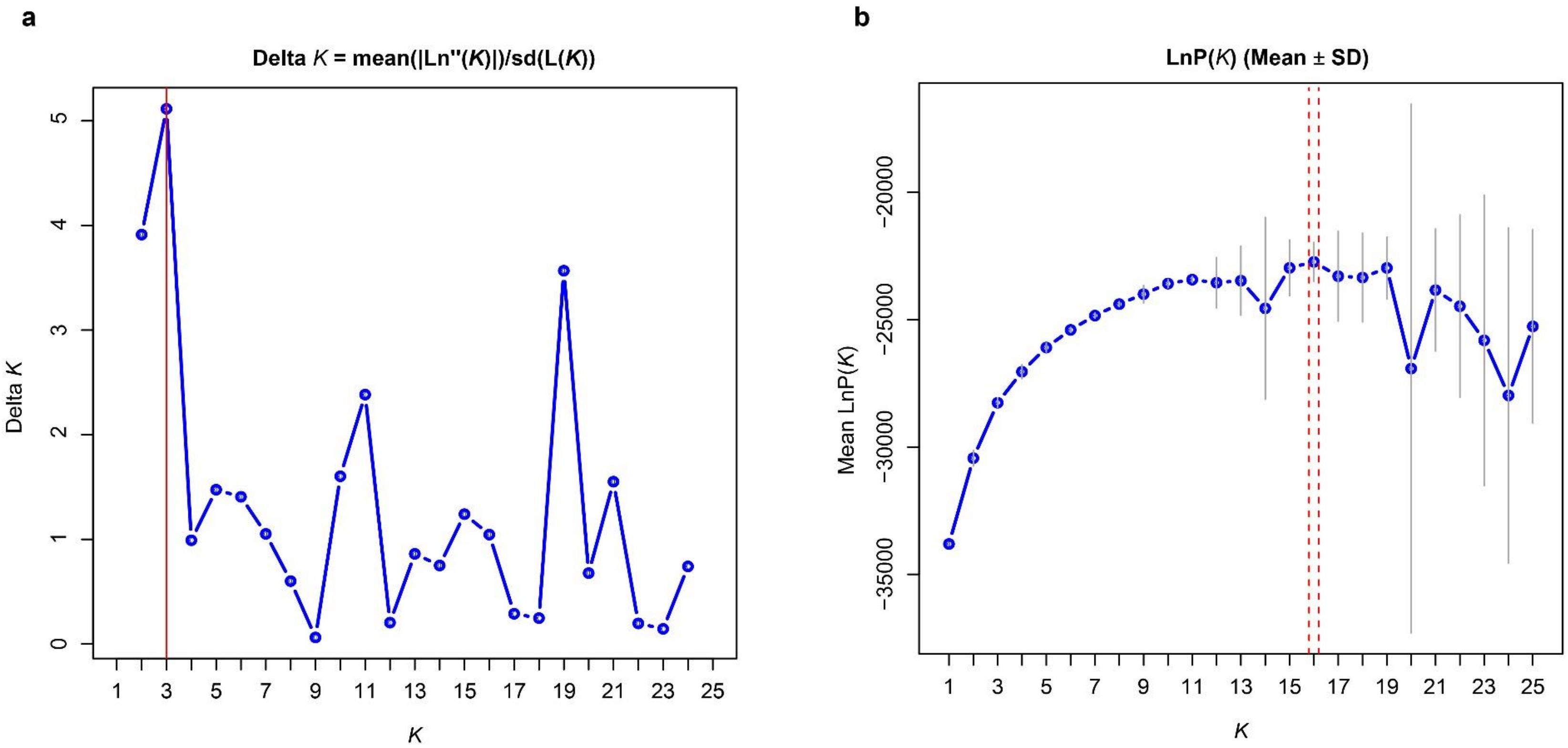

Supplement 14. Different population structure patterns of Korean chicken varieties, generated by model-based Bayesian clustering algorithms implemented in STRUCTURE. (a) Plot of Evanno's Δ*K* and (b) Plot of LnP(*K*)
