# Supplementary material for: Divergent ancestry of Korean native and Thai chickens with independent gene pool retention by Korean commercial chickens
Source: Anim Biosci. 2025 Oct 22;39(3):250315. doi: 10.5713/ab.25.0315 (PMC12963744; doi:10.5713/ab.25.0315)
Supplement: Supplementary file 15 [file ab-25-0315-Supplementary-15.pdf]

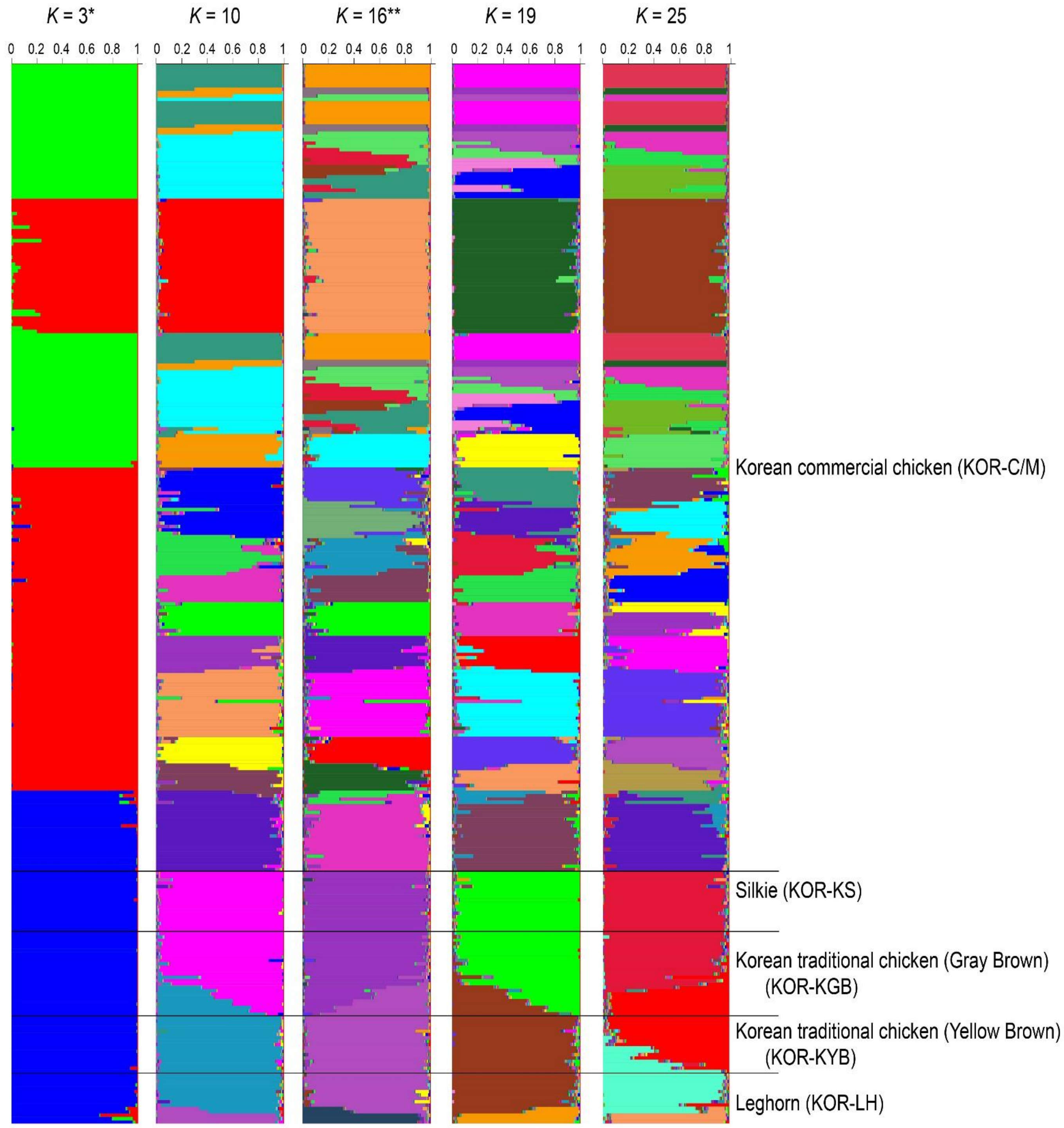

Supplement 15. Population structure of five Korean chicken varieties. The *x*-axis represents the proportion of membership (posterior probability) in each genetic cluster, while each horizontal bar on the *y*-axis represents an individual. All individuals from the five varieties are superimposed on the plot. Black vertical lines indicate the boundaries. The highest posterior probability, denoted by *, was determined based on Evanno's Δ*K*, and the highest ln P(*K*) is represented by **
