# Supplementary material for: Divergent ancestry of Korean native and Thai chickens with independent gene pool retention by Korean commercial chickens
Source: Anim Biosci. 2025 Oct 22;39(3):250315. doi: 10.5713/ab.25.0315 (PMC12963744; doi:10.5713/ab.25.0315)
Supplement: Supplementary file 16 [file ab-25-0315-Supplementary-16.pdf]

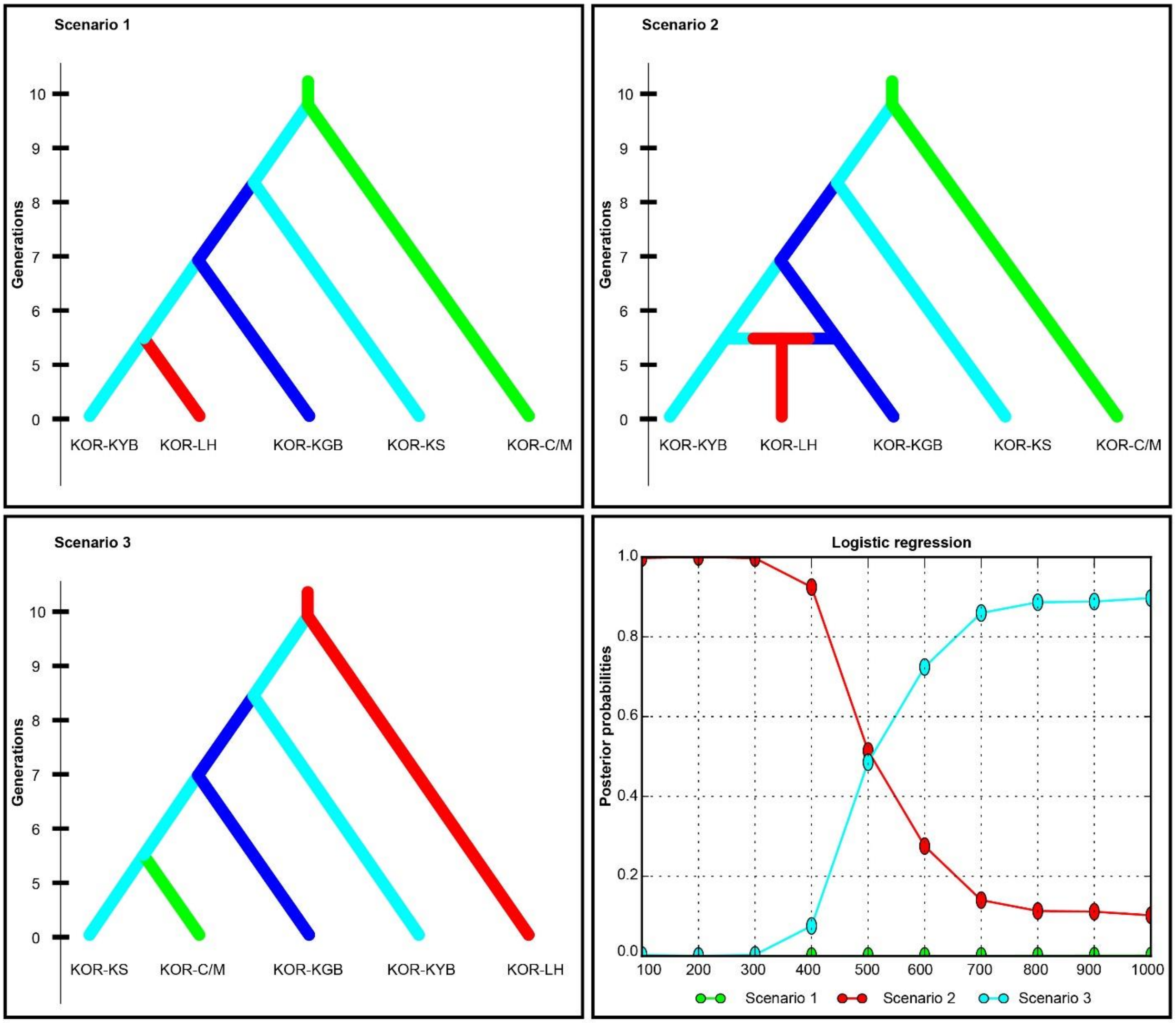


**Supplement 16.** Assessment of population history models for the Korean chicken varieties using approximate Bayesian computation (ABC) inference
