# Supplementary material for: Divergent ancestry of Korean native and Thai chickens with independent gene pool retention by Korean commercial chickens
Source: Anim Biosci. 2025 Oct 22;39(3):250315. doi: 10.5713/ab.25.0315 (PMC12963744; doi:10.5713/ab.25.0315)
Supplement: Supplementary file 18 [file ab-25-0315-Supplementary-18.pdf]

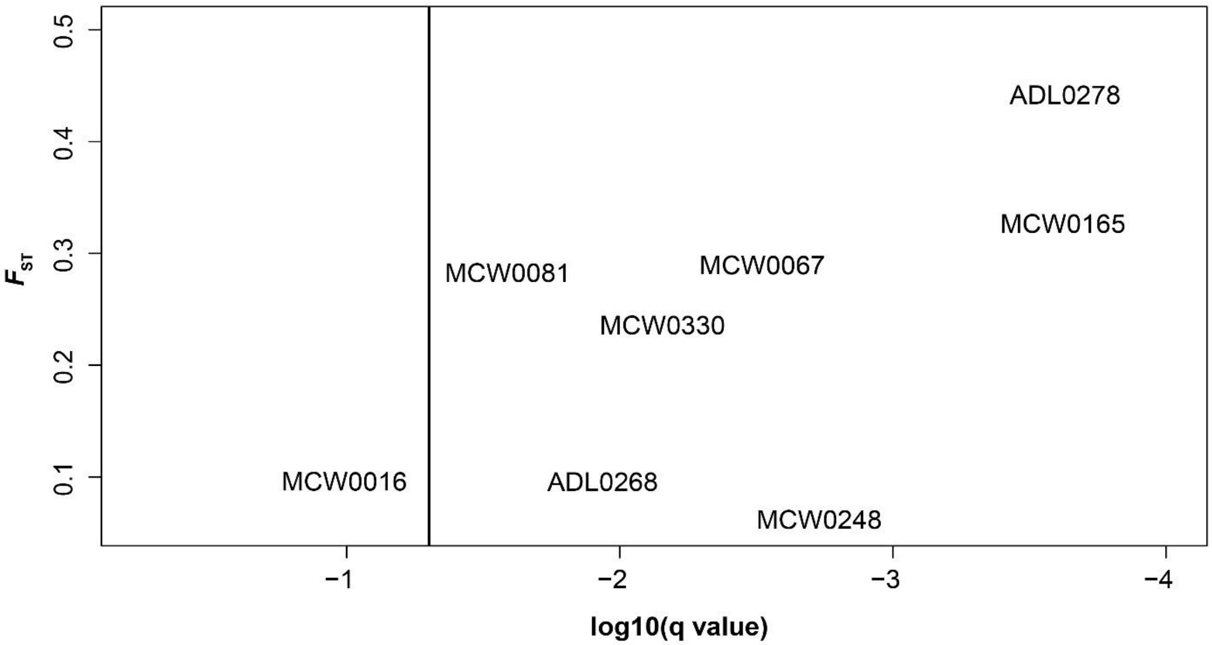

Supplement 18. BAYESCAN estimate for the probability of a locus under selection. Results of F_{ST} values for each locus are plotted against their log-transformed Bayes factor
