# Supplementary material for: Divergent ancestry of Korean native and Thai chickens with independent gene pool retention by Korean commercial chickens
Source: Anim Biosci. 2025 Oct 22;39(3):250315. doi: 10.5713/ab.25.0315 (PMC12963744; doi:10.5713/ab.25.0315)
Supplement: Supplementary file 19 [file ab-25-0315-Supplementary-19.pdf]

**Supplement 19.** Wilcoxon sign rank test to evaluate mutation–drift equilibrium in 315 Korean chickens under different models. Data were calculated using Bottleneck version 1.2.02 (Piry et al., 1999).

| Varieties | Wilcoxon test | | Mode-shift test |
|---|---|---|---|
| | TPM | SMM | |
| | Probability for one tail test for *H* excess | Probability for one tail test for *H* excess | |
| KOR-C/M | 1.000 | 1.000 | normal L-shaped distribution |
| KOR-KS | 0.124 | 0.257 | normal L-shaped distribution |
| KOR-KGB | 0.761 | 0.878 | normal L-shaped distribution |
| KOR-KYB | 0.151 | 0.214 | normal L-shaped distribution |
| KOR-LH | 0.401 | 0.584 | normal L-shaped distribution |

KOR-C/M = Korean commercial chicken; KOR-KS = Silkie; KOR-KGB = Korean traditional chicken (Gray Brown); KOR-KYB = Korean traditional chicken (Yellow Brown); KOR-LH = Leghorn (LH)

Piry, S.; Luikart, G.; Cornuet, J.M. BOTTLENECK: a program for detecting recent effective population size reductions from allele data frequencies. J. Hered. 1999, 90, 502–503. doi: 10.1093/jhered/90.4.502
