# Supplementary material for: Divergent ancestry of Korean native and Thai chickens with independent gene pool retention by Korean commercial chickens
Source: Anim Biosci. 2025 Oct 22;39(3):250315. doi: 10.5713/ab.25.0315 (PMC12963744; doi:10.5713/ab.25.0315)
Supplement: Supplementary file 20 [file ab-25-0315-Supplementary-20.pdf]

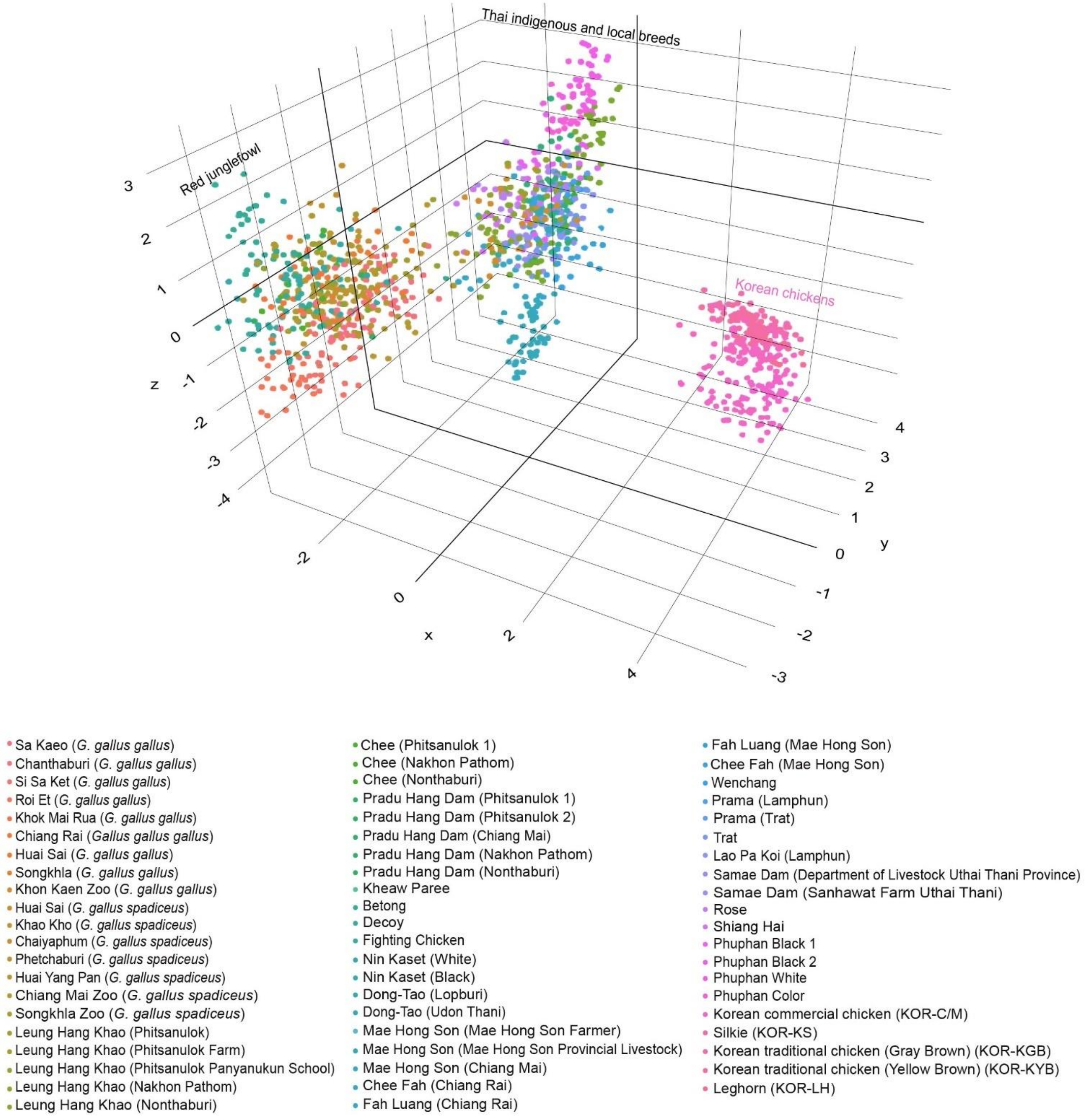

Supplement 20. Discriminant Analysis of Principal Components (DAPC) of Korean, Thailand local and indigenous chicken varieties, and red junglefowl. Different colours indicate scatter plots based on DAPC output for assigned genetic clusters. Dots represent different individuals
